# Supplementary figures and images for: Inhibition of lysosomal LAMTOR1 increases autophagy by suppressing the MTORC1 pathway to ameliorate lipid accumulations in MAFLD
Source: Autophagy. 2025 Jul 6;21(12):2633–49. doi: 10.1080/15548627.2025.2519054 (PMC12758200; doi:10.1080/15548627.2025.2519054)

**Figure 2.**

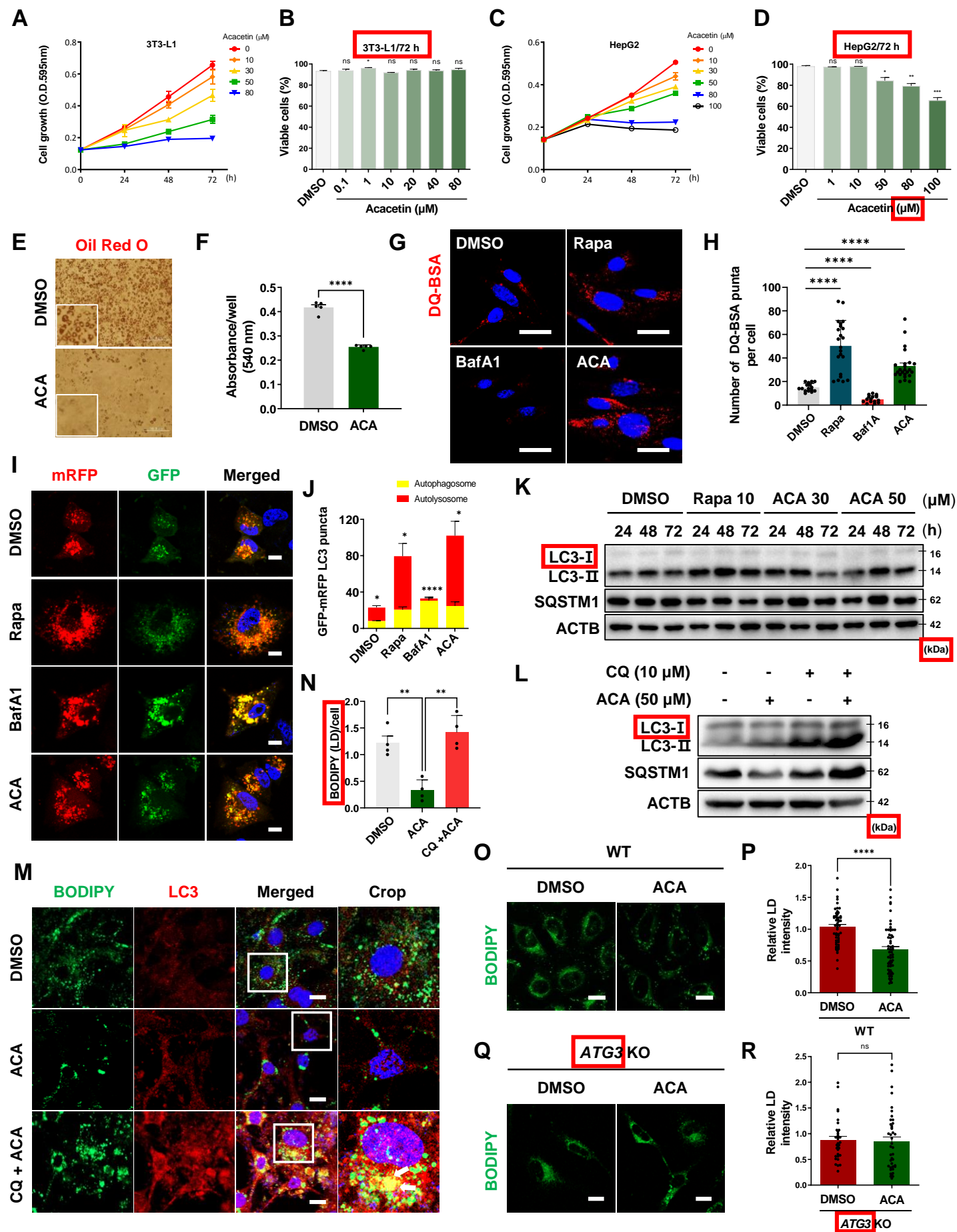

Supplement: Figure 2_red_250528.pdf [file KAUP_A_2519054_SM3944.pdf]

Figure 7.

A

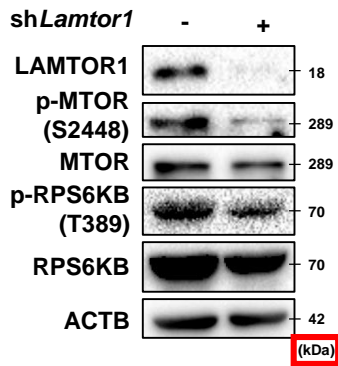

B

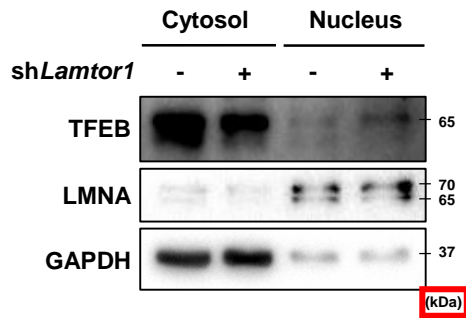

C

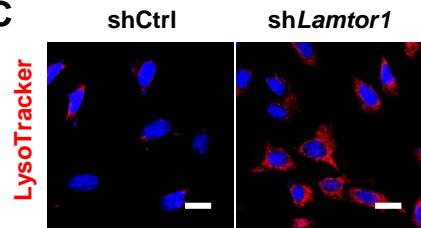

D

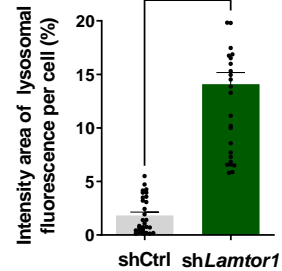

E

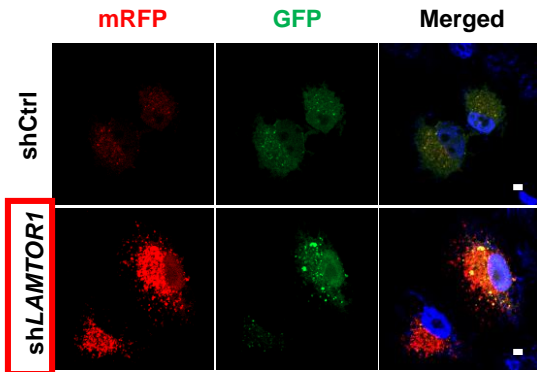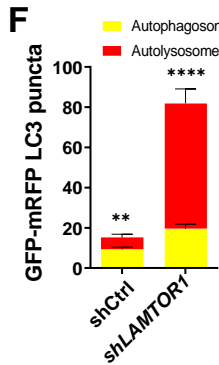

G

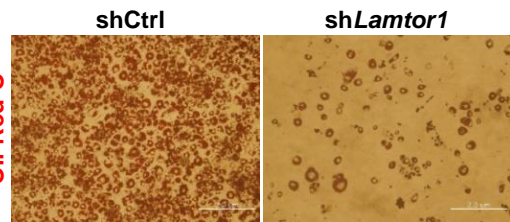

H

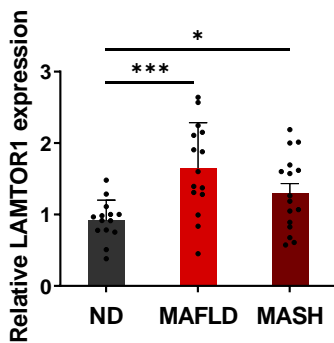

I

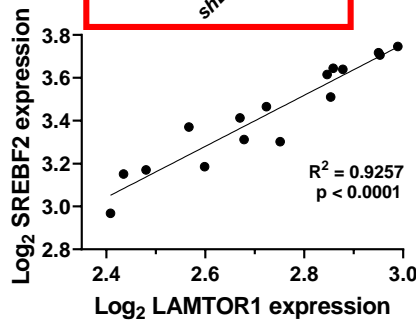

J

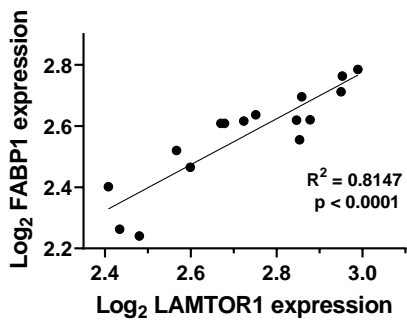

K

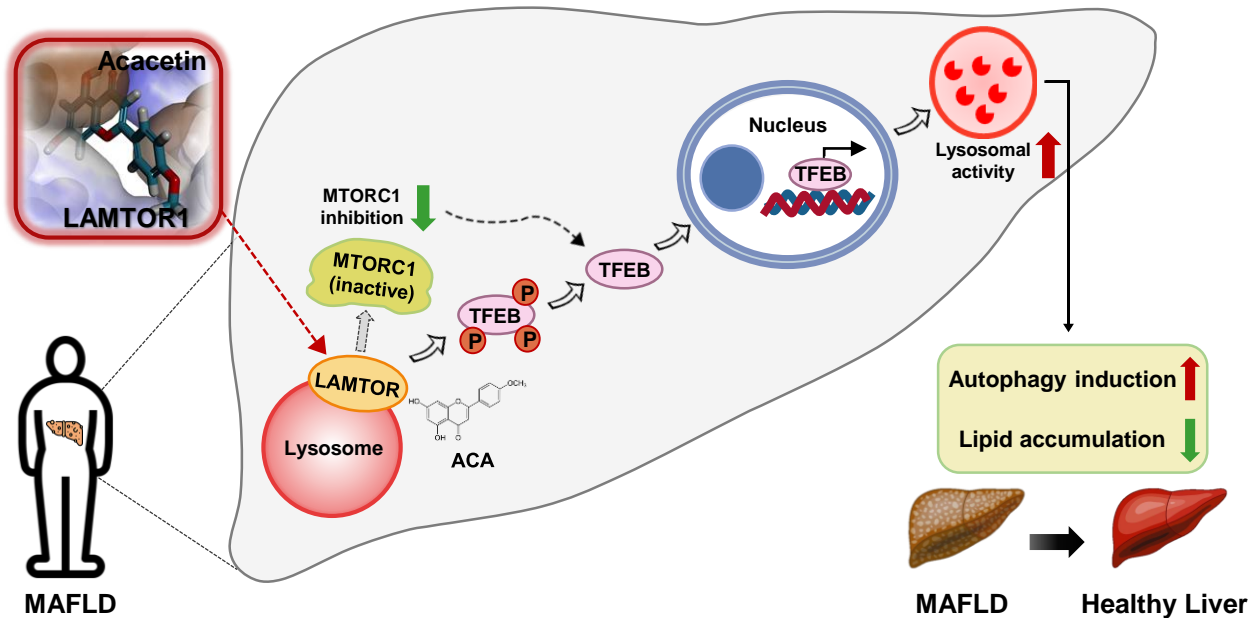

Supplement: Figure 7_red_250528.pdf [file KAUP_A_2519054_SM3943.pdf]

Figure 3.

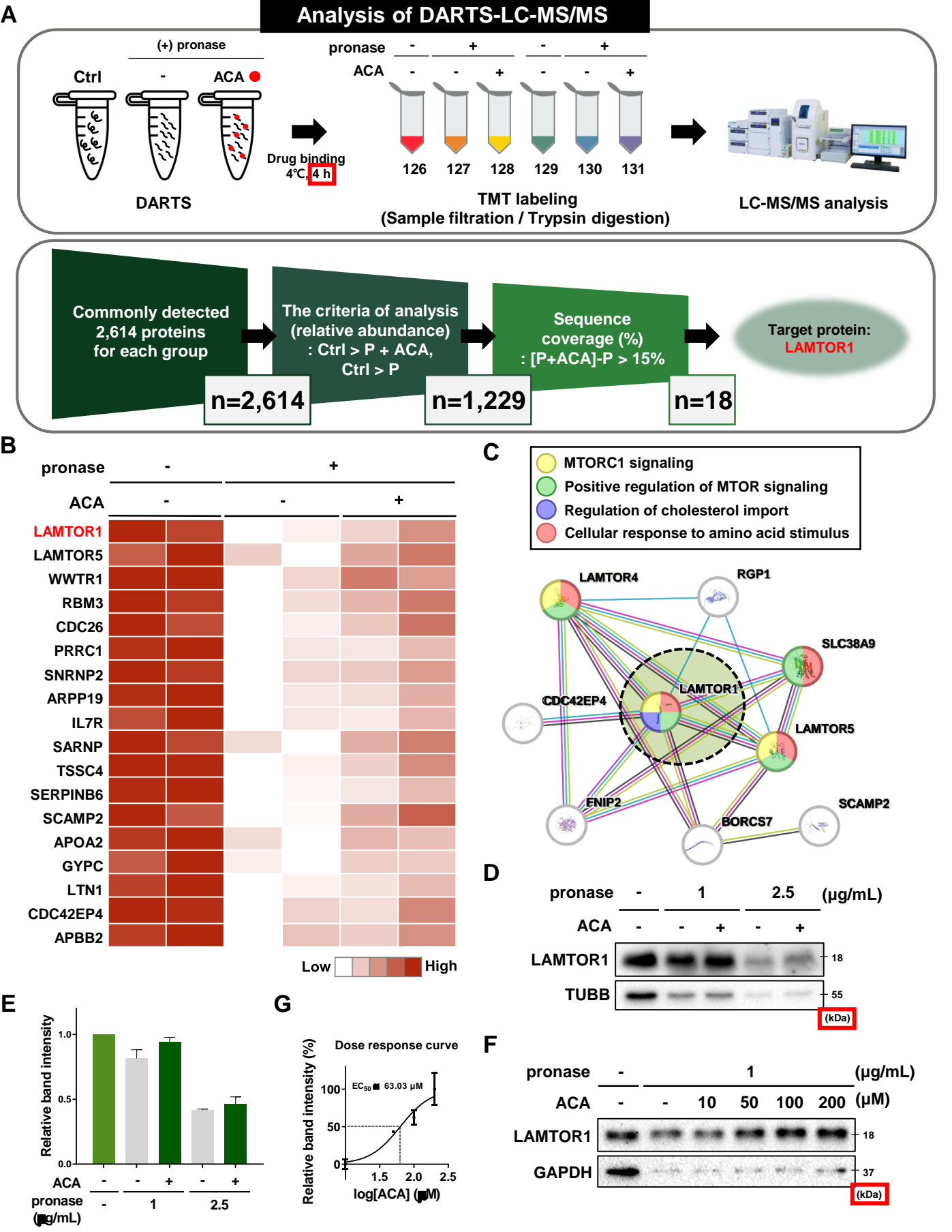

Supplement: Figure 3_red_250528.pdf [file KAUP_A_2519054_SM3941.pdf]

Figure 5.

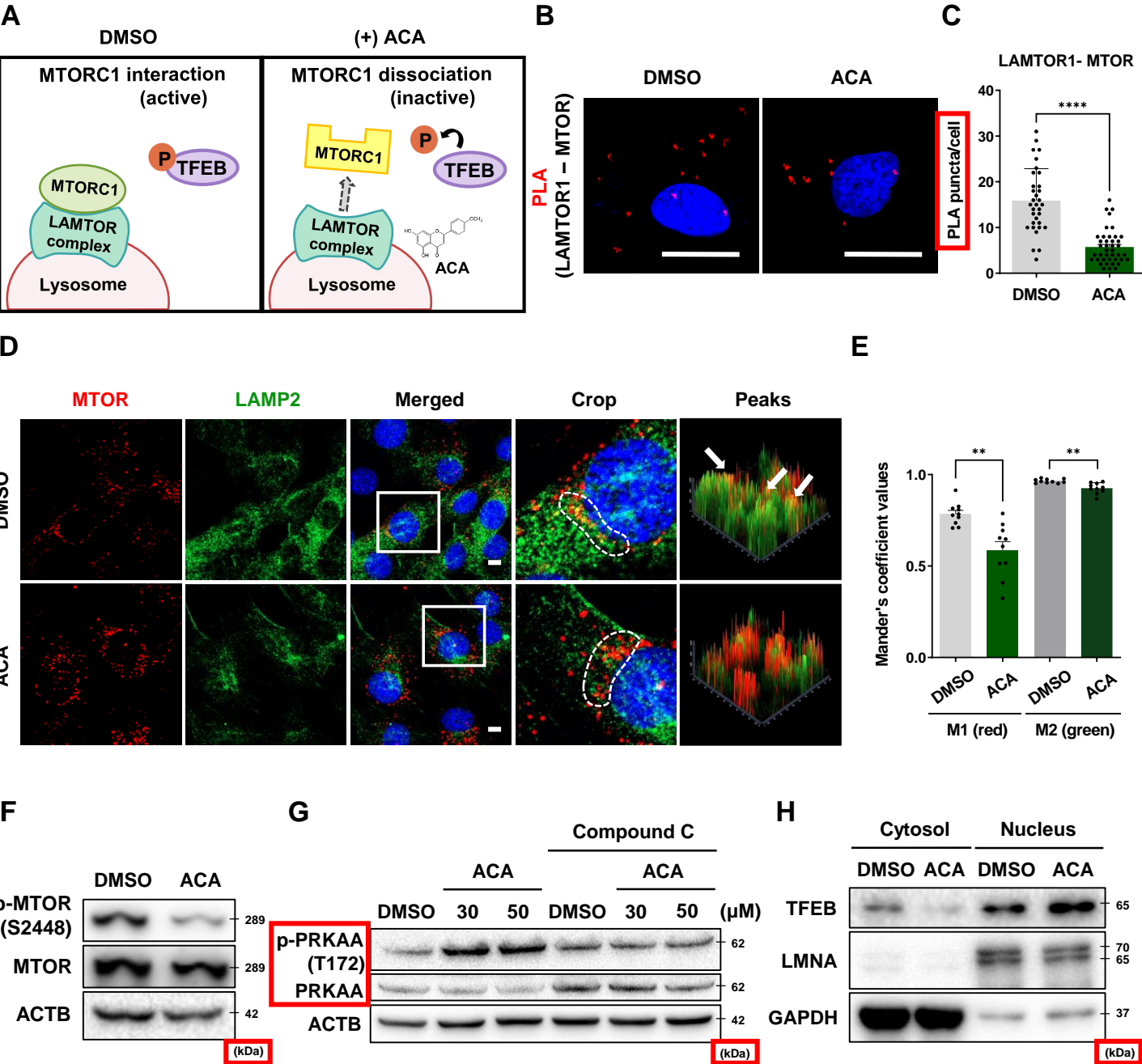

Supplement: Figure 5_red_250528.pdf [file KAUP_A_2519054_SM3940.pdf]

Figure 6.

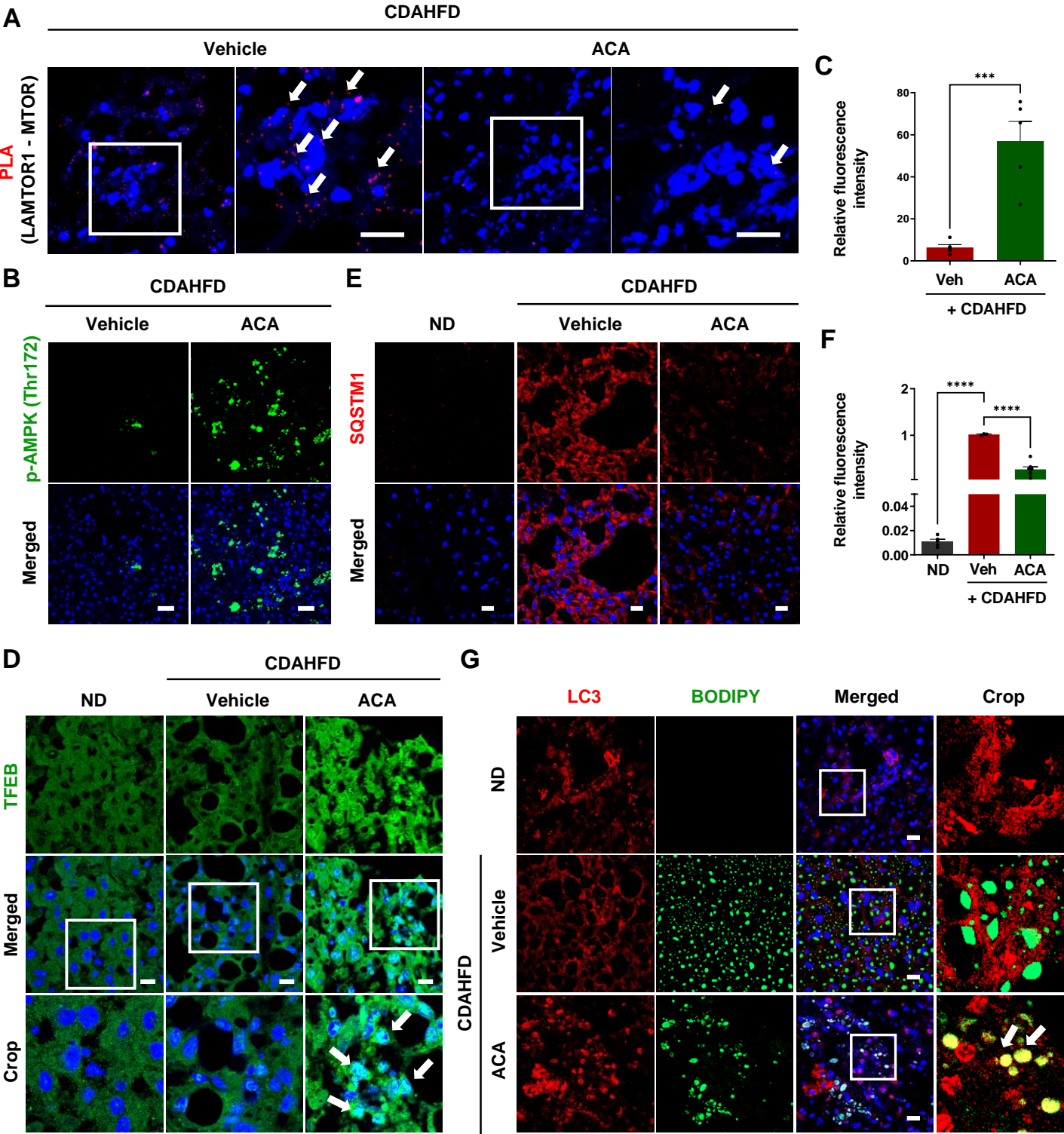

Supplement: Figure 6_red_250528.pdf [file KAUP_A_2519054_SM3938.pdf]

**Figure 4.****A**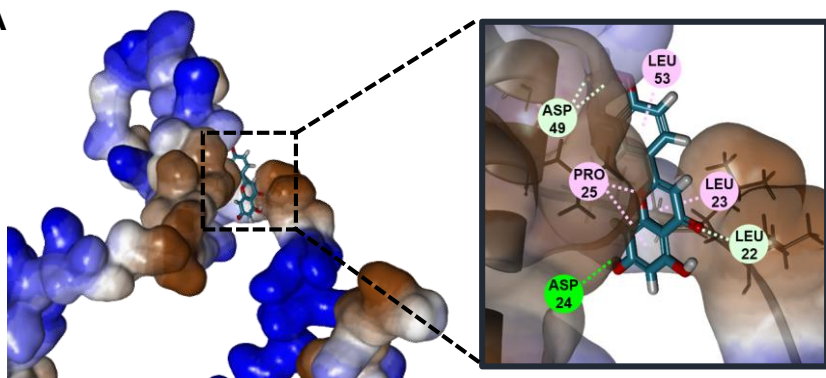**B**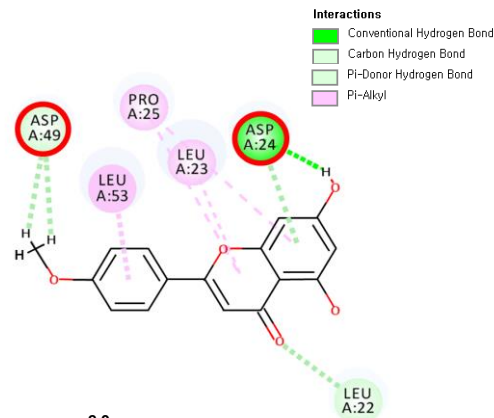**C**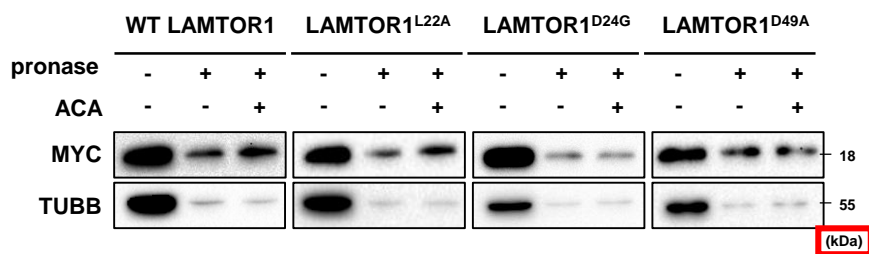**D**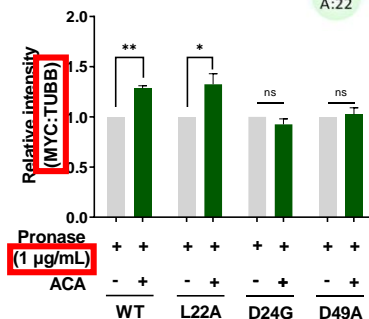**E**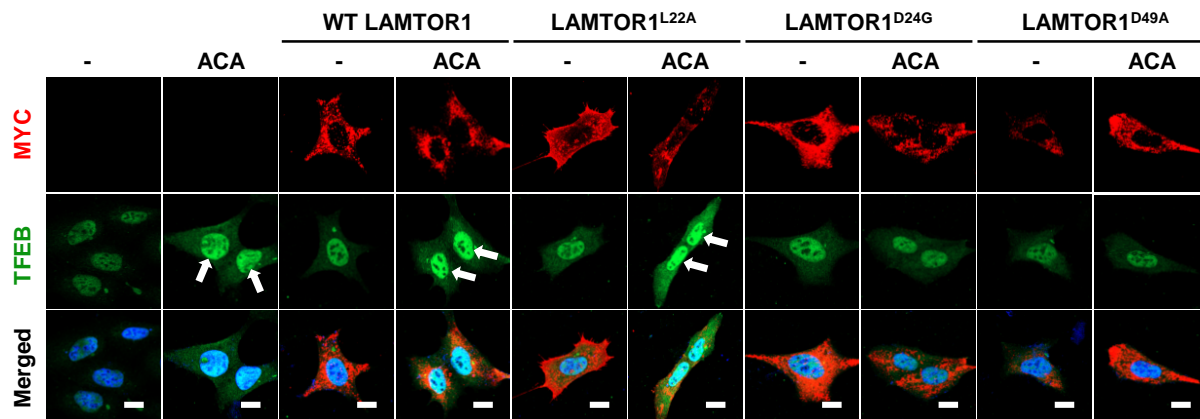**F**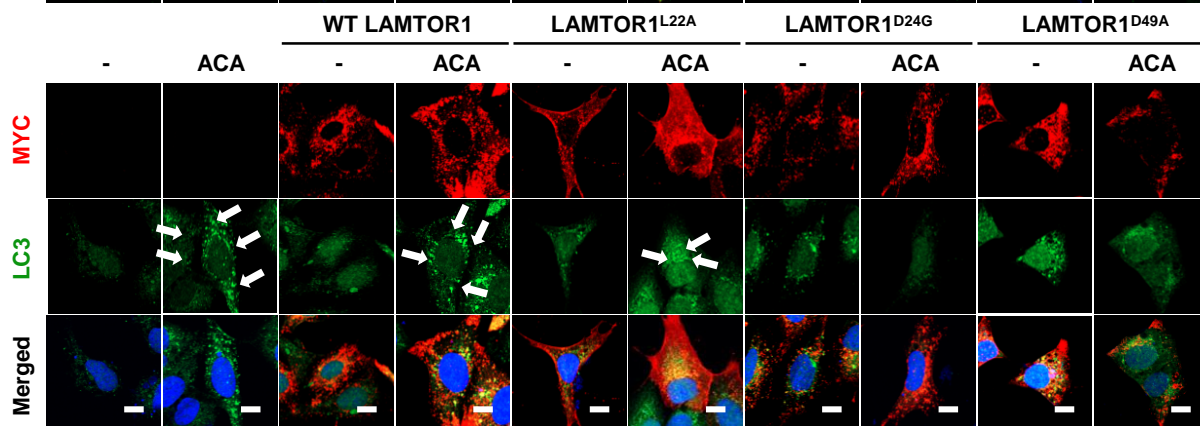**G**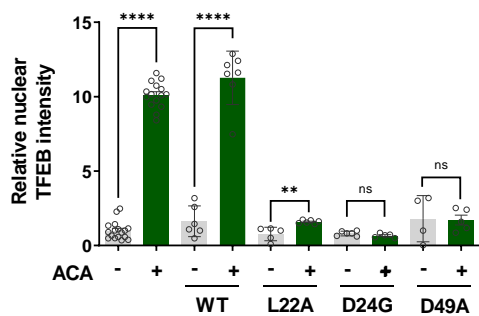**H**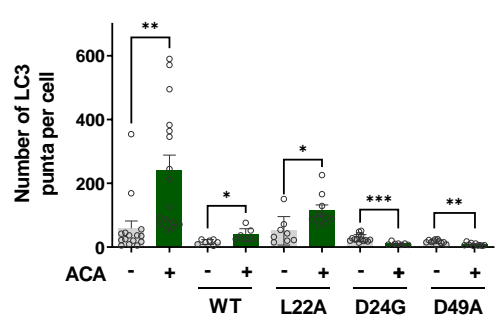

Supplement: Figure 4_red_250528.pdf [file KAUP_A_2519054_SM3937.pdf]

**Figure 1.**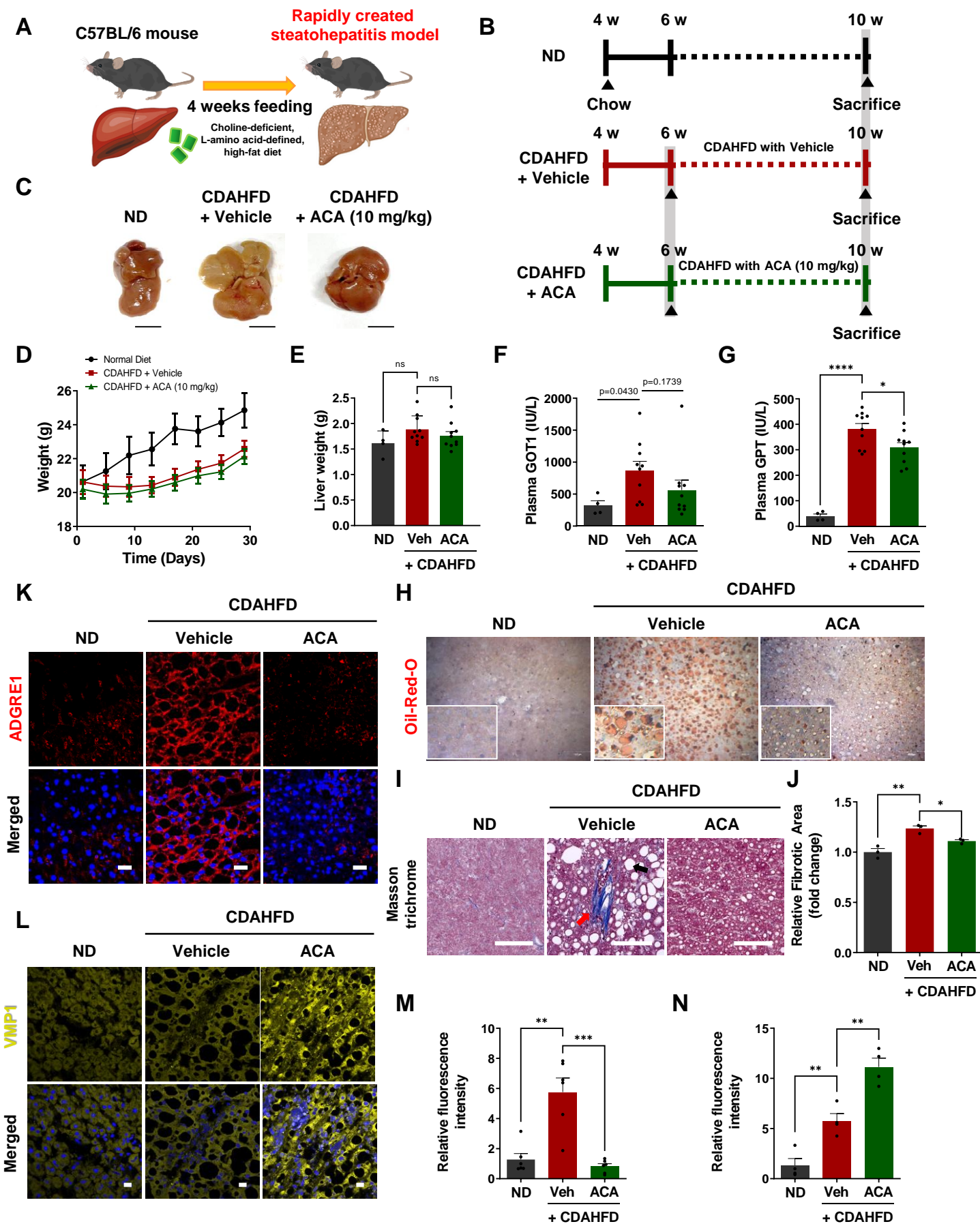

Supplement: Figure 1_red_250528.pdf [file KAUP_A_2519054_SM3934.pdf]
