# Supplementary material for: Inhibition of lysosomal LAMTOR1 increases autophagy by suppressing the MTORC1 pathway to ameliorate lipid accumulations in MAFLD
Source: Autophagy. 2025 Jul 6;21(12):2633–49. doi: 10.1080/15548627.2025.2519054 (PMC12758200; doi:10.1080/15548627.2025.2519054)
Supplement: acacetin_MS_not for review_R3_250528.docx [file KAUP_A_2519054_SM3939.docx]

**Inhibition of Lysosomal LAMTOR1 Increases Autophagy by Suppressing the MTORC1 Pathway to Ameliorate Lipid Accumulations in MAFLD**

Yunyeong Jang^a^, Minjeong Ko^a^, Ju Yeon Lee^b,c^, Jin Young Kim^b,c^, Eun-Woo Lee^d,e^ and Ho Jeong Kwon^a*^

^a^*Chemical Genomics Leader Research Laboratory,* *Department of Biotechnology, College of Life Science & Biotechnology, Yonsei University, Seoul 03722,* *Republic of Korea.*

^b^*Digital Omics Research Center, Korea Basic Science Institute, Ochang 28119, Republic of Korea.*

^c^*Critical Diseases Diagnostics Convergence Research Center, Korea Research Institute of Bioscience and Biotechnology, Daejeon 34141, Republic of Korea*

^d^*Metabolic Regulation Research Center, Korean Research Institute of Bioscience and Biotechnology (KRIBB), Daejeon 34141, Republic of Korea.*

*^e^School of Pharmacy, Sungkyunkwan University, Suwon 16419, Republic of Korea.*

**Contact** Ho Jeong Kwon [kwonhj@yonsei.ac.kr](mailto:kwonhj@yonsei.ac.kr) Department of Biotechnology, College of Life Science & Biotechnology, Yonsei University, 50 Yonsei-ro, Seodaemun-gu, Seoul 03722, Republic of Korea

**Abstract**

Metabolic dysfunction-associated fatty liver disease (MAFLD) is a serious metabolic disorder characterized by fat accumulation in the liver, which can trigger liver inflammation and fibrosis, potentially leading to cirrhosis or liver cancer. Despite many studies, effective treatments for MAFLD remain elusive due to its complex etiology. In this study, we have focused on the discovery of therapeutic agents and molecular targets for MAFLD treatment. We demonstrated that the natural compound acacetin (ACA) alleviates MAFLD by regulating macroautophagy/autophagy in a CDAHFD mouse model of rapidly induced steatohepatitis. In addition, ACA inhibits lipid accumulation in 3T3-L1 adipocytes through autophagy induction. To identify the target responsible for the autophagy activity induced by ACA, we performed drug affinity responsive target stability (DARTS) combined with LC-MS/MS proteomic analysis. This led to the identification of LAMTOR1 (late endosomal/lysosomal adaptor, MAPK and MTOR activator 1), a lysosomal membrane adaptor protein. We found that binding of ACA to LAMTOR1 induces its release from the LAMTOR complex, leading to inhibition of MTOR (mechanistic target of rapamycin kinase) complex 1 (MTORC1), thereby increasing autophagy. This process helps ameliorate metabolic disorders by modulating the MTORC1-AMPK axis. Genetic knockdown of LAMTOR1 phenocopies the effects of ACA treatment, further supporting the role of LAMTOR1 as a target of ACA. These findings suggest LAMTOR1 plays a crucial role in ACA's therapeutic effects on MAFLD. In summary, our study identifies LAMTOR1 as a key protein target of ACA, revealing a potential therapeutic avenue for MAFLD by modulating autophagy via the LAMTOR1-MTORC1-AMPK signaling pathway.

Keywords: Acacetin; autophagy; DARTS; LAMTOR1; MAFLD; MTORC1

**Abbreviations**

ACA: acacetin; ADGRE1/EMR1/F4/80: adhesion G protein-coupled receptor E1; CDAHFD: choline-deficient amino acid-defined, high-fat diet; CETSA: cellular thermal shift assay; CQ: chloroquine; DARTS: drug affinity responsive target stability; DQ-BSA: dye quenched-bovine serum albumin; GOT1/AST: glutamic-oxaloacetic transaminase 1; GPT/ALT: glutamic--pyruvic transaminase; LAMP2: lysosomal associated membrane protein 2; LAMTOR1: late endosomal/lysosomal adaptor, MAPK and MTOR activator 1; LC-MS/MS: liquid chromatography-tandem mass spectrometry; MAFLD: metabolic dysfunction-associated fatty liver disease; MAP1LC3B/LC3: microtubule associated protein 1 light chain 3 beta; MASH: metabolic dysfunction-associated steatohepatitis; mRFP-GFP-MAP1LC3B: tandem fluorescent-tagged MAP1LC3B; MTORC1: mechanistic target of rapamycin complex 1; PA: palmitic acid; PRKAA/AMPK: AMP-activated protein kinase; PLA: proximity ligation assay; Rapa: rapamycin; RPS6KB1/p70S6K: ribosomal protein S6 kinase B1; RRAG: Ras related GTP binding; SQSTM1: sequestosome 1; TFEB: transcription factor EB; VMP1: vacuole membrane protein 1.

**Introduction**

Metabolic dysfunction-associated fatty liver disease (MAFLD) is characterized by excessive fat accumulation in the liver, independent of alcohol consumption, which can lead to hepatocyte injury, inflammation, liver fibrosis, and ultimately, hepatocellular carcinoma [1]. MAFLD is strongly associated with several metabolic disorders, including obesity and diabetes, and its global prevalence is increasing, making it a major health concern [2-5]. However, there are currently no FDA-approved treatments for MAFLD or related liver diseases [6], emphasizing the urgent need for novel therapeutic agents and molecular targets for its treatment.

Autophagy, a cellular process that removes of damaged organelles and proteins, is a critical mechanism for metabolic regulation. Autophagy enables cells to regulate lipid metabolism to maintain cellular energy homeostasis. Dysfunction of autophagy is closely linked to the development of MAFLD [6-10]. Previous studies have shown that enhancing autophagy can suppress hepatic steatosis, whereas impaired autophagy accelerates the progression of MAFLD. As a result, several strategies are being explored to alleviate MAFLD by promoting autophagy [11], although the precise mechanisms behind autophagy induction remain poorly understood.

Flavonoids, a group of bioactive compounds, have long been recognized for their anti-inflammatory, antioxidant, antimicrobial, and autophagy-inducing properties. One such flavonoid, acacetin (ACA; 5,7-dihydroxy-4’methoxyflavone), extracted from the Korean mint (*Agastache rugosa*), is widely used for both culinary and medicinal purposes. ACA exhibits various biological activities including antioxidant, anti-inflammatory, anticancer, and lipid metabolism regulation [12,13]. Previous studies have highlighted the protective effects of ACA against MAFLD, demonstrating its ability to regulate AMP-activated protein kinase (AMPK) expression, modulate endoplasmic reticulum stress, and inhibit ferroptosis, ultimately reducing lipid accumulation [14,15]. Despite these promising effects, the specific mechanisms by which ACA regulates autophagy to alleviate MAFLD have not been fully addressed. Notably, ACA has been identified as an autophagy inducer in other diseases, modulating autophagy to alleviate disease progression [14,16,17]. Based on these findings, we hypothesize that ACA may improve MAFLD by regulating autophagy.

To identify the target protein of label-free ACA and understand its mechanism of action, we performed drug affinity responsive target stability (DARTS) combined with liquid chromatography with tandem mass spectrometry (LC-MS/MS) analysis [18-20]. This approach led to the identification of LAMTOR1 (late endosomal/lysosomal adaptor, MAPK and MTOR activator 1) as a key target protein that binds to ACA and undergoes structural changes, resulting in increased resistance to proteolysis. As a member of the LAMTOR complex located on the lysosomal membrane, LAMTOR1 interacts with RRAG (Ras related GTP binding) proteins to regulate the activation of MTOR (mechanistic target of rapamycin kinase) complex 1 (MTORC1) [21,22]. LAMTOR1 plays a critical role in the regulation of autophagy in MAFLD by modulating lysosomal function and lipid accumulation. Our results reveal a mechanism by which ACA alleviates lipid disorders by targeting LAMTOR1 and inducing autophagy through modulation of the MTORC1-AMPK axis, providing a novel therapeutic target and approach for MAFLD.

**Results**

***Acacetin ameliorates pathological liver damage in the CDAHFD-induced MASH mouse model.***

We first investigated whether ACA could ameliorate liver injury in a mouse model of metabolic-associated steatohepatitis (MASH) induced by CDAHFD. The CDAHFD-induced MASH mouse model causes hepatic steatosis by impairing the secretion of hepatic VLDL-triglyceride, leading to inflammation and fibrosis, which closely mimics the pathology of human MAFLD [23].

ACA or a vehicle control was administered via intraperitoneal injection every other day for 4 weeks (**Figure 1A-1C**). There was no significant difference in body or liver weights between the vehicle and ACA-treated group (**Figure 1D and 1E**). Both GOT1/AST and GPT/ALT showed a decreasing trend, although the reduction in GOT1/AST in the ACA-treated group was not statistically significant due to an outlier. On average, however, GOT1/AST levels were reduced by 35% in the ACA-treated group compared to the vehicle-treated group (**Figure 1F and 1G**).

Oil Red O staining revealed a significant reduction in lipid accumulation in the liver of the ACA-treated group compared to the vehicle-treated group (**Figure 1H**). Masson’s trichrome staining demonstrated a decrease in fibrotic areas in the liver tissue of the ACA-treated group (**Figure 1I and 1J**), suggesting that ACA has protective effects against chronic liver injury. Immunofluorescence staining showed that the ACA-treated group exhibited a reduction in the expression of ADGRE1/EMR1, a marker of hepatic macrophages recruited during MASH (**Figure 1K and 1M**). In addition, VMP1, an endoplasmic reticulum transmembrane protein suppressed in MASH [24], was decreased in CDAHFD mice but was restored in the ACA-treated group (**Figure 1L and 1N**). These results demonstrated that ACA is a promising natural compound for ameliorating liver injury in the MASH mouse model by regulating lipid metabolism and inflammation.

***Acacetin inhibits lipid accumulation in an autophagy-dependent manner.***

Based on the observed efficacy of ACA in the MASH mouse model (**Figure 1**), we further investigated its mode of action in the 3T3-L1 (adipocytes) and HepG2 (hepatocellular carcinoma) cell lines, which are widely used for *in vitro* studies of MAFLD. To determine the appropriate concentration of ACA for each cell line, we conducted an MTT proliferation assay. The IC_50_ values at 72 h were approximately 34 µM for the 3T3-L1 cells and 40 µM for HepG2 cells (**Figure 2A and 2C**). In addition, the trypan blue assay showed no significant effect on cell viability up to 80 µM ACA treatment (**Figure 2B and 2D**). Notably, a significant reduction in lipid accumulation was observed by Oil Red O staining when differentiated 3T3-L1 cells were treated with 50 µM ACA (**Figure 2E and 2F**).

Emerging evidence suggests that autophagy plays an important role in various cellular processes, including lipid metabolism, insulin sensitivity, and the pathogenesis of MAFLD [11]. However, it remains unclear whether ACA regulates lipid metabolism through autophagy in MAFLD. To evaluate the autophagic activity of ACA, we performed a dye quenched-bovine serum albumin (DQ-BSA) assay to detect lysosomal-specific digestion in 3T3-L1 cells. Rapamycin (Rapa), an autophagy inducer, increases DQ-BSA fluorescence [25], whereas the autophagy inhibitor bafilomycin A_1_ decreases DQ-BSA fluorescence. Similarly, ACA increased DQ-BSA fluorescence (**Figure 2G and 2H**), suggesting that ACA induces autophagic activity.

In HepG2 cells transiently expressing mRFP-GFP-MAP1LC3B/LC3, ACA increased the mRFP:GFP ratio, indicating an increased autophagic flux (**Figure 2I and 2J**). In differentiated 3T3-L1 cells, western blot analysis showed that ACA induced the conversion of LC3-Ⅰ to LC3-Ⅱ and promoted SQSTM1 degradation in a time-dependent manner, similar to the effects of Rapa, demonstrating that ACA effectively stimulates autophagic flux (**Figure 2K**). Notably, LC3-II levels increased up to 48 h after ACA treatment but decreased at 72 h. This reduction suggests that the autophagic process was not impaired but had progressed to completion, with LC3-II being degraded via lysosomal activity. Co-treatment with the autophagy inhibitor chloroquine led to a further accumulation of LC3-II, suggesting that ACA promotes autophagic flux in 3T3-L1 cells (**Figure 2L)**. In contrast, co-treatment with the proteasome inhibitor MG132 and ACA did not affect ACA-induced SQSTM1 degradation (**Figure S1**), indicating that ACA promotes protein degradation through the autophagic pathway rather than the proteasome-dependent pathway. To assess whether autophagy contributes to the lipid-lowering effects of ACA, we co-treated cells with ACA and CQ and measured lipid levels using BODIPY staining. Consistent with the results in **Figure 2E**, ACA reduced BODIPY fluorescence intensity, but this effect was suppressed when CQ was added, indicating that the lipid-lowering effect of ACA is autophagy-dependent (**Figure 2M and 2N**).

In wild-type (WT) cells, ACA treatment resulted in a reduction in BODIPY fluorescence intensity **(Figures 2O and 2P)**. However, in *ATG3* knockout (KO) cells, which impair autophagosome formation and exhibit autophagy deficiency, ACA treatment did not alter BODIPY fluorescence intensity **(Figures 2Q and 2R)**. These observations further support the conclusion that the lipid-lowering effect of ACA is mediated through autophagy.

***Identification of the pharmacological target of acacetin by DARTS LC-MS/MS analysis.***

To investigate the mechanism by which ACA induces autophagy and alleviates MAFLD, we employed a label-free target protein identification method, Drug Affinity Responsive Target Stability (DARTS), combined with LC-MS/MS [26].

Membrane and cytosolic protein fractions were obtained from 3T3-L1 adipocytes. Each protein pool was treated with either vehicle control or ACA, followed by pronase digestion. TMT labeling and LC-MS/MS analysis were then performed to identify and quantify the proteome in the samples.

Among a total of 2,614 membrane proteins detected, 18 candidate proteins exhibited more than a 15% increase in sequence coverage in ACA-treated samples compared to controls, as determined by LC-MS/MS. This increase in sequence coverage suggests enhanced resistance to pronase digestion (**Figure 3A and 3B**). Using STRING analysis, we explored the network between these candidate target proteins and their partners, revealing a network primarily associated with MTORC1 signaling, cholesterol import regulation, and cellular response to amino acid stimulation (**Figure 3C**).

LAMTOR components are key proteins involved in the regulation of MTORC1 signaling. Based on DARTS LC-MS/MS analysis, ACA bound to both LAMTOR1 and LAMTOR5, with LAMTOR1 showing a higher resistance to pronase degradation (log_2_ fold change of 0.35) compared to LAMTOR5 (log_2_ fold change of 0.24). This suggests that ACA stabilizes LAMTOR1 more effectively than LAMTOR5.

LAMTOR1 anchors directly to the lysosomal membrane and regulates cellular functions by controlling MTORC1 activation and lipid signaling [22]. It also stabilizes components of the Ragulator-RRAG complex and modulates autophagy [27]. Therefore, we focused on LAMTOR1 as a target candidate linked to the autophagy activity of ACA.

We confirmed that ACA binds to LAMTOR1 and increased its stability against pronase degradation through DARTS western blot validation (**Figure 3D and 3E**). The binding affinity between LAMTOR1 and ACA increased in a dose-dependent manner, with an EC_50_ value of approximately 44 µM (**Figure 3F and 3G**), which was consistent with the concentration that was effective for cell proliferation (**Figure 2A and 2C**).

The binding of ACA to LAMTOR1 was further validated using CETSA, another label-free binding assay that assesses changes in the thermal stability of the protein due to compound binding [28]. ACA was shown to reduce the heat-induced denaturation of LAMTOR1 in the temperature range of 40°C to 55°C, suggesting that ACA binds to LAMTOR1 and induces its structural changes that make it more sensitive to thermal denaturation (**Figure S2A and S2C**). Furthermore, the isothermal dose-response CETSA showed that ACA dose-dependently reduced heat-induced denaturation of LAMTOR1 (**Figure S2B and S2D**). Taken together, our results suggest that ACA regulates autophagy through its direct binding to LAMTOR1.

***Analysis of the binding site of acacetin to LAMTOR1 by* in silico *docking.***

To identify the binding site of ACA on LAMTOR1, we performed an *in silico* docking analysis using the AlphaFold structure of LAMTOR1 (ID: Q9CQ22). The results showed a binding energy of -16.7921 kcal/mol (**Figure 4A**), with ACA forming conventional hydrogen bonds with D24, non-conventional hydrogen bonds with L22 and D49, and hydrophobic interactions with L23, P25, and D53 in LAMTOR1 (**Figure 4B**).

Since both conventional and non-conventional hydrogen bonds significantly affect binding interactions [29], we generated point mutants for L22, D24, and D49 to identify the key residues involved in ACA binding to LAMTOR1. These residues were selected based on their ability to form either conventional hydrogen bonds (e.g., involving electronegative atoms like oxygen or nitrogen) or non-conventional hydrogen bonds (e.g., carbon-hydrogen bonds). We transfected HEK293 cells with MYC-tagged wild-type (WT) or mutant (L22A, D24G, or D49A) LAMTOR1 plasmids.

The DARTS assay demonstrated that ACA treatment resulted in a 44.9% increase in stability for WT LAMTOR1 and a 35.7% increase for LAMTOR1^L22A^. However, the stability of the LAMTOR1^D24G^ and LAMTOR1^D49A^ remained unchanged after ACA treatment (**Figure 4C and 4D**). In addition, in cells expressing WT LAMTOR1 or LAMTOR1^L22A^, ACA treatment promoted nuclear translocation of TFEB and increased LC3 levels, an autophagy marker (**Figure 4E-H and Figure S3**). In contrast, in cells expressing the LAMTOR1^D24G^ or LAMTOR1^D49A^ mutants, the nuclear translocation of TFEB was not significantly observed, and LC3 levels were reduced (**Figure 4E-H and Figure S3**). These findings collectively suggest that residues D24 and D49 are critical for ACA binding to LAMTOR1 and its autophagy-inducing activity.

***Acacetin induces TFEB nuclear translocation by modulating the MTORC1-AMPK axis.***

We next explored how ACA induces autophagy through LAMTOR1. The lysosomal membrane contains several protein complexes that are critical for cellular processes. Among them, the Ragulator is a multi-protein complex that includes LAMTOR1 to LAMTOR5, acting as a coactivator for MTORC1 and AMPK, integrating signaling within lysosomes in response to nutrients and energy levels [30]. The V-ATPase proton pump also interacts with Ragulator, promoting the activation of RRAG and MTORC1 [31,32].

When MTORC1 is activated, it interacts with the Ragulator complex, anchoring MTORC1 to the lysosomal surface, which suppresses autophagy [33]. However, when MTORC1 dissociates from the Ragulator complex, it promotes the activation of autophagy (**Figure 5A**) [21]. Upon ACA treatment, the interaction between LAMTOR1 and MTOR was significantly reduced, as demonstrated by a decrease in red fluorescence signals in the proximity ligation assay (PLA) conducted in 3T3-L1 cells (**Figure 5B and 5C**). Similarly, ACA treatment reduced the colocalization of LAMP2 (a lysosomal marker) and MTOR, further indicating that the inhibition of MTORC1 (**Figure 5D and 5E**). Additionally, ACA treatment led to a decrease in MTOR phosphorylation in differentiated 3T3-L1 cells (**Figure 5F**).

As previously reported, ACA also induced a dose-dependent increase in AMPK phosphorylation in these cells (**Figure 5G**) [14]. Importantly, the activation of AMPK by ACA was completely blocked by compound C, an AMPK inhibitor (**Figure 5G**). Both the inhibition of MTORC1 and activation of AMPK are known to activate the TFEB signaling pathway [34-36]. Consistent with this, we observed that ACA treatment increased the nuclear translocation of TFEB in 3T3-L1 cells (**Figure 5H**), suggesting that ACA induces autophagy through modulation of the MTORC1-AMPK axis.

***Acacetin restores autophagy in the MASH mouse model.***

Palmitic acid (PA)-treated HepG2 cells are commonly used to study MAFLD *in vitro* [37]. In the context of PA-induced metabolic dysfunction, we observed the accumulation of LC3 and SQSTM1, indicating impaired autophagic flux. However, treatment with ACA successfully restored autophagic flux (**Figure S4A**). Notably, when CQ was co-treated with PA and ACA, the autophagic flux was inhibited, preventing ACA from restoring autophagy, as evidenced by the accumulation of SQSTM1 (**Figure S4A**).

In the mouse model, the CDAHFD diet led to the accumulation of LC3 and SQSTM1 in liver tissue, signifying impaired autophagy. ACA treatment activated autophagy in the CDAHFD-fed control group by promoting the conversion of LC3-I to LC3-II and facilitating the degradation of SQSTM1 (**Figure S4B**). Consistent with our *in vitro* findings, PLA indicated that the interaction between LAMTOR1 and MTOR was reduced in the liver tissues of the ACA-treated group (**Figure 6A**). Additionally, ACA treatment induced AMPK activation through increased phosphorylation of AMPK (**Figure 6B and 6C**). The nuclear translocation of TFEB was also observed in the ACA-treated group (**Figure 6D**). The accumulation of SQSTM1, which indicates autophagy dysregulation in the CDAHFD-fed control group, was alleviated in the ACA-treated group (**Figure 6E and 6F**). Furthermore, the colocalization of BODIPY and LC3 was enhanced in the ACA-treated group, demonstrating that ACA promoted autophagy to degrade lipid droplets (**Figure 6G**). Taken together, these results demonstrated that ACA activates autophagy by regulating LAMTOR1, thereby alleviating the pathological autophagy dysregulation associated with MASH.

***LAMTOR1 inhibition contributes to lipid reduction via autophagy and is as an important factor in MAFLD and MASH patients.***

We next investigated the role of LAMTOR1 in autophagy and lipid metabolism by gene knockdown analysis. In LAMTOR1 knockdown cells, we observed a downregulation of the phosphorylation levels of MTOR and RPS6KB1 (**Figure 7A**). In addition, the nuclear translocation of TFEB was increased in 3T3-L1 cells (**Figure 7B**), along with increased lysosomal activity and autolysosome formation (**Figure 7C-7F**). We further examined the effect of LAMTOR1 knockdown on lipid metabolism. Oil Red O staining of differentiated 3T3-L1 cells revealed a reduction of in lipid droplets following LAMTOR1 knockdown (**Figure 7G**). These results suggest that the inhibition of LAMTOR1 promotes lipid reduction through autophagy, supporting that ACA targets LAMTOR1 to exert its lipid-lowering effects.

Additionally, we analyzed LAMTOR1 expression in liver samples from patients with MAFLD (including MASH) using publicly available human MAFLD RNA-seq datasets. Notably, analysis revealed that *LAMTOR1* mRNA levels were significantly higher in MAFLD or MASH patients compared to healthy individuals (**Figure 7H**) [38]. Previous studies have shown that the mRNA levels of *SREBF2* (sterol regulatory element binding transcription factor 2) and *FABP1* (fatty acid binding protein 1), both associated with the development and progression of MAFLD and MASH, are also upregulated in the MAFLD and MASH patient groups [39-42]. This suggests that LAMTOR1 may serve as a potential risk factor for the development of liver disease in humans (**Figure 7I-J**).

**Discussion**

In this study, we identified LAMTOR1 as the target of ACA, a natural compound that regulates metabolic disorders through autophagy. Recent studies have highlighted the direct association between autophagy and the development and progression of MAFLD [43]. However, the molecular mechanisms regulating autophagy in MAFLD remain largely unclear, which underscores the importance of therapeutic approaches targeting autophagy for MAFLD.

To identify the protein target of ACA, we used DARTS LC-MS/MS analysis, which allows the identification of target proteins without chemical or structural modification of the compound [44]. Using this innovative method, we also evaluated the direct interaction between ACA and LAMTOR1 and assessed its stable binding affinity using CDOCKER energy in an *in silico* docking analysis. This analysis revealed that ACA forms hydrogen bonds with D24 and D49 (LAMTOR1^D24G^ and LAMTOR1^D49A^), which led to reduced pronase resistance. Additionally, the suppression of autophagy induction by ACA was observed when the D24 and D49 mutants of LAMTOR1 were expressed in HEK293 cells. These results suggest that the N-terminal aspartic acid residues D24 and D49 of LAMTOR1 play critical roles in ACA-induced autophagy regulation (**Figure 4E-H and Figure S3**). Given the limited structural information for LAMTOR1, future studies will focus on elucidating the functional roles of these residues and on determining the crystal structure of LAMTOR1. Furthermore, although our data suggest a potential interaction between ACA and LAMTOR1, additional studies are required to confirm direct binding. These findings underscore the need for further biophysical investigations, which will be pursued in future work to better characterize the ACA-LAMTOR1 interaction.

The lysosomal Ragulator complex (LAMTOR1 to LAMTOR5) is pivotal in regulating the MTORC1 signaling pathway [45]. Within this complex, LAMTOR1 stabilizes the interaction between the LAMTOR2-LAMTOR3 and LAMTOR4-LAMTOR5 roadblock domain heterodimers, anchoring them to the lysosomal membrane (**Figure S5A and S5B**) [46]. DARTS LC-MS/MS analysis also identified LAMTOR5 as a candidate target for ACA in addition to LAMTOR1. DARTS western blot analysis further confirmed that ACA binds to LAMTOR5 (**Figure S5C**). However, LAMTOR5 exhibited lower resistance to proteolytic degradation than LAMTOR1 and is present at lower levels in cells. Therefore, we focused on LAMTOR1 as the primary protein target of ACA. Interestingly, our results suggest for the first time that ACA interacts not only with LAMTOR1 but also with other components of Ragulator complexes (LAMTOR2 to LAMTOR5). This may influence MTORC1 regulation through conformational changes in the Ragulator-RRAG complex. Given the complexity of the Ragulator-RRAG complex, further studies are needed to fully elucidate the mechanism by which ACA affects MTORC1 signaling through this interaction.

The biological activity of small molecules often involves the modulation of multiple cellular targets. Therefore, we cannot exclude the potential contributions of other candidate target proteins identified by the DARTS LC-MS/MS analysis to the autophagy-inducing effects of ACA. In this study, we used DARTS LC-MS/MS analysis to identify the target protein of ACA and demonstrated that while LAMTOR1 plays a critical role in regulating autophagy, it remains important to investigate other proteins involved in autophagy. A more systematic exploration of other potential autophagy-related proteins will be a key focus in our future research.

Excessive activation of MTORC1 due to dysregulation has been implicated in several diseases, including aging, diabetes, osteoporosis, cancer, and neurological disorders [47]. MTORC1 inhibitors have been explored as therapeutic agents for MAFLD and MASH. However, clinical trials have not led to the approval of these inhibitors due to limitations such as rebound activation of autophagy, which can counteract their therapeutic effects. Direct MTORC1 inhibitors, such as torin-1 and Rapa, effectively suppresses MTORC1 activity by binding directly to the complex. However, sustained inhibition of MTORC1 can disrupt essential cellular processes, including protein synthesis, energy metabolism, and cell growth, potentially leading to adverse effects such as muscle atrophy, immunosuppression, and neurodegeneration [48-51]. Chronic MTORC1 inhibition can also suppress MTORC2, impairing Akt signaling and cell survival [50,52], while activating compensatory pathways such as PI3K/Akt and MAPK, reducing therapeutic efficacy and promoting drug resistance [52,53].

In conclusion, we identified LAMTOR1 as a biologically relevant target for ACA in the reduction of metabolic disorders. Mechanistically, ACA binds directly to LAMTOR1, inhibiting MTORC1 and induction of autophagy. This study highlights the critical role of LAMTOR1 in metabolic regulation and suggests that targeting LAMTOR1 may represent a promising strategy for metabolic disorders, including MALFD (**Figure 7K**).

**Materials and Methods**

***Cell culture and treatment***

3T3-L1 preadipocytes (ATCC, CL-173) were cultured in DMEM (Gibco, 11995-065) supplemented with 10% bovine serum (Gibco, 16170-078) and 1% penicillin-streptomycin (Gibco, 15140-122). To induce differentiation of 3T3-L1 preadipocytes into adipocytes, cells were cultured in MDI medium containing 3-isobutyl-1-methylxanthine (Sigma-Aldrich, I5879), dexamethasone (Sigma-Aldrich, D8893), and insulin (Sigma-Aldrich, I0516) for 2 days, followed by incubation in insulin-containing medium for an additional 2 days. The medium was then supplemented with 10% FBS (Gibco, 16000-044) and 1% antibiotics (Gibco, 15240-062). HepG2, HEK293, and HeLa cells were obtained from the Korean Cell Line Bank (KCLB). Cells were cultured in DMEM (Gibco, 11995-065) supplemented with 10% FBS (Gibco, 16000-044) and 1% antibiotics. All cell cultures were maintained in a humidified incubator at 37°C in a 5% CO_2_ atmosphere.

***MTT assay***

To evaluate the effect of ACA (Sigma-Aldrich, 00017) on cell proliferation, cells were treated with ACA and incubated for 24 to 72 h. Cell proliferation was evaluated using the MTT colorimetric assay. MTT (VWR International, LLC, 0793), dissolved in PBS (CureBio, P0213; diluted in distilled water), was added to the cells for 3 h, resulting in a final concentration of 0.4 mg/mL. The MTT formazan product was dissolved in dimethyl sulfoxide (Sigma-Aldrich, D2650), and the absorbance was measured at 540 nm using a microplate reader (BioTek Instruments, Winooski, VT, USA).

***Cell viability assay***

Cell viability and cytotoxicity were assessed using trypan blue staining (Sigma-Aldrich, T8154) 72 h after treatment with ACA.

***Western blot analysis***

For protein extraction from cells or tissues, soluble proteins were harvested by addition of SDS lysis buffer (50 mM Tris-HCl, pH 6.8 containing 10% glycerol, 2% SDS, 10 mM dithiothreitol, and 0.005% bromophenol blue). Proteins were separated by SDS-PAGE and transferred to a PVDF membrane. Blots were blocked and immunolabeled overnight at 4°C with the following primary antibodies: LC3B (Cell Signaling Technology, 2775s; Abcam, ab48394), SQSTM1 (Abcam, ab56416), LAMTOR1 (Cell Signaling Technology, 8975s), LAMTOR5 (Cell Signaling Technology, 14633s), MYC (Medical & Biological Laboratories, M192-3), p-MTOR (Cell Signaling Technology, 5536s), MTOR (Invitrogen, 215Q18), p-PRKAA/AMPK (Cell Signaling Technology, 2535s), AMPK (Abcam, ab110036), p-RPS6KB (Cell Signaling Technology, 9206s), RPS6KB (Cell Signaling Technology, 9202s), TFEB (Cell Signaling Technology, 32361s; Proteintech, 13372-1-AP), LMNA (Santa Cruz Biotechnology, sc20681), TUBB (Abcam, ab6046), ACTB (Abcam, ab8226), and GAPDH (Cell Signaling Technology, 2118s). Western blot images were acquired using Image Lab software version 6.1 and quantified using ImageJ software.

***Drug Affinity Responsive Target Stability (DARTS)***

3T3-L1 cells were harvested and lysed using Mem-PER membrane protein extraction lysis buffer (Thermo Fisher Scientific, 89842). Samples were diluted to a total protein concentration of 1.5 mg/mL and incubated with vehicle control (DMSO) or ACA for 3 h at 4 °C on a rotator. Pronase (Sigma-Aldrich, 10165921001) was added and incubated on a rotator for 10 min at room temperature, and then samples were boiled to stop the pronase reaction. The processed samples were subsequently used for western blot and LC-MS/MS analysis.

***Sample preparation and TMT labeling***

DARTS samples were denatured with 8 M urea (Sigma-Aldrich, U-5378). To reduce disulfide bonds, samples were reacted with 500 mM TCEP (Tris[2-carboxyethyl]phosphine; Sigma-Aldrich, C4706) for 1 h at room temperature. Alkylation was then performed by adding 500 mM iodoacetamide (Sigma-Aldrich, I1149) and incubating for 1 h at room temperature in the dark. The alkylated protein solution was then replaced with 200 mM triethylammonium bicarbonate buffer (Sigma-Aldrich, T7408), and the protein solution was concentrated using a 10 K centrifugal filter (MilliporeSigma, UFC5010). Total protein concentration was measured using the BCA protein assay (Thermo Fisher Scientific, 23227). Proteins were then treated with MS grade trypsin (1:40 [w/w]; Promega, V5280) and digested at 37°C for 16 h. According to the manufacturer’s instructions, the trypsin-digested peptides were labeled with a 6-plex TMT reagent (Thermo Fisher Scientific, 90061) for 1 h at room temperature. The reaction was quenched by the addition of 5% hydroxylamine solution (Sigma-Aldrich, 467804). Samples were dried with a speed vacuum and stored at −80°C until analysis.

***High pH reversed-phase liquid chromatography for peptide fractionation***

The dissolved TMT 6-plex-labeled sample was fractionated on a Nexera XR HPLC (Shimadzu, Kyoto, Japan) using an XBridge BEH Shield RP18 column (130 Å, 2.5 μm, 4.6 × 150 mm, Waters) with a 70-min gradient from 5% to 95% mobile phase B at a flow rate of 1.0 mL/min. Mobile phases A and B consisted of 5 mM ammonium formate in 100% water and 5 mM ammonium formate in 95% acetonitrile, respectively; both buffers were adjusted to a pH of 10 with ammonium hydroxide. A total of 40 fractions were collected using an FRC-10A fraction collector (Shimadzu, Kyoto, Japan), after starting the elution with an interval of 1 min for each fraction. The 40 fractions were concentrated into 10 fractions. The pooled fractions were dried and stored at −80°C freezer until LC-MS/MS analysis.

***LC-MS/MS analysis***

TMT-labeled samples were analyzed using an Easy-nLC 1200 (Thermo Fisher Scientific, Germering, Germany) and an Orbitrap Fusion Lumos mass spectrometer (Thermo Fisher Scientific, San Jose, CA, USA) equipped with a nano-electrospray source. An autosampler was used to load peptide solutions onto a C_18_ trap column with an internal diameter (ID) of 75 μm, length of 20 mm, and particle size of 3 μm (Thermo Fisher Scientific). Peptides were desalted and concentrated on the trap column for 10 min at a flow rate of 4 μL/min, and separated on the analytical C_18_ column (75 μm × 50 cm, Thermo Fisher Scientific) with the mobile phases consisting of 100% water (A) and 80% acetonitrile (B), each containing 0.1% formic acid at 250 nL/min. The liquid chromatography gradient started with 5% mobile phase B and was maintained for 5 min. Mobile phase B was ramped linearly to 38% for 90 min, and 95% for 5 min. 95% mobile phase B was maintained for 9 min before decreasing to 5% for another 1 min. The column was re-equilibrated with 5% mobile phase B for 10 min prior to the next run. The voltage applied to generate the electrospray was 2.0 kV. During the chromatographic separation, the Orbitrap Fusion Lumos was operated in data-dependent mode, automatically switching between MS1 and MS2 with a 3 s cycle time. MS data were acquired using the following parameters: full scans (400-200 m/z) were acquired with a maximum ion injection time of 100 ms at a resolution of 120,000 in the Orbitrap and automatic gain control (AGC) target value of 2.0× 10^5^. MS/MS spectra were acquired from 110 m/z at 60,000 resolution, with a high energy collision dissociation (HCD) of 37.5% normalized collision energy within 1.4 Da isolation window. AGC target value was 5.0 × 10^4^ with maximum ion injection time of 118 ms. The exclusion time for previously fragmented ions was 30 s within 10 ppm at the Korea Basic Science Institute.

***Protein identification and quantitation***

The Integrated Proteomics Pipeline with built-in search engines (IP2, version 6.5.5, Integrated Proteomics Applications, Inc, CA, USA) was used for data analysis with the UniProt mouse protein database (September 2020, reviewed 17,045 proteins). The inverted sequences of all proteins were appended to the database for false discovery rate (FDR) calculation. ProLucid was used to identify the peptides [54], with a precursor mass error of 5 ppm, and a fragment ion mass error of 200 ppm. Trypsin was selected as the enzyme, with two potential missed cleavages. TMT modification (+229.1629) at the N terminus and lysine residues by the labeling reagent and carbamidomethylation at cysteine were chosen as static modifications. Oxidation at methionine was chosen as the variable modification. Reporter ions were extracted from small windows (±20 ppm) around their expected m/z in the HCD spectrum. The output data files were filtered and sorted to generate the protein list using the DTASelect (The Scripps Research Institute, San Diego, CA, USA) with two and more peptide assignments for a protein identification and a false positive rate of less than 0.01 [55].

Quantitative analysis was performed using Census in the IP2 pipeline (IP2, version 6.5.5, Integrated Proteomics Applications, Inc, San Diego, CA, USA) with only the unique peptides. The intensity at a reporter ion channel for a protein was calculated as the average of the intensities of that reporter ion from all constituent peptides of the identified protein [56]. Reverse and potentially spurious proteins were removed. Protein intensities averaged from the reporter ion intensities of all identified TMT-labeled peptides were uploaded onto the Perseus platform (version 1.6.14.0). The data were normalized by subtracting the median values based on columns after being transformed as log_2_ values. An ANOVA test was performed to select significant proteins below 0.05 p-value between groups.

***Cellular Thermal Shift Assay (CETSA)***

Live cells were harvested with trypsin-EDTA solution. The cells were then treated with vehicle control (DMSO) or 50 μM ACA in a CO_2_ incubator at 37°C for 1 h with gentle shaking every 15 min. The cells were subsequently centrifuged, washed with PBS, and resuspended in PBS supplemented with protease inhibitor cocktail (Thermo Fisher Scientific, 1861282). The suspension was aliquoted into PCR tubes, heated for 3 min in a PCR thermal cycler (Bio-Rad Laboratories, Hercules, CA, USA), and then cooled to 25°C. The pellet was resuspended in Membrane Protein Extraction Lysis Buffer, frozen twice in liquid nitrogen, and then centrifuged at 20,000 *g* for 20 min at 4°C. Soluble proteins were collected and subjected to western blot analysis.

***Lipid droplet staining***

Differentiated 3T3-L1 adipocytes were treated with drugs for the indicated period of time, fixed with 4% formaldehyde (Sigma-Aldrich, 252549), washed with PBS, and stained with 0.3% Oil Red O solution (Sigma-Aldrich, O0625) for 1 h at room temperature. The samples were washed with PBS and images were captured using a light microscope. After imaging, the cells were dried overnight at room temperature, and dye extraction buffer (Sigma-Aldrich, I9516) was added. The absorbance of the extracted Oil Red O dye solution was measured at 460 nm using a microplate reader (BioTek Instruments, Winooski, VT, USA).

***Transfection***

For tandem LC3 analysis, cells were transfected with the mRFP-GFP-MAP1LC3B/LC3 plasmid, which was kindly provided by Prof. Jaewhan Song (Yonsei University, Seoul, Republic of Korea), using Lipofectamine 3000 (Invitrogen, L3000015) according to the manufacturer's instructions. For gene knockdown or overexpression studies, cells were transfected with the sh*LAMTOR1* plasmid (OriGene, TL513263) or *LAMTOR1* (MYC-DDK-tagged) clone (OriGene, MR201262) using Lipofectamine 3000 according to the manufacturer's instructions.

***Immunofluorescence staining***

Cells were treated with drugs, fixed with 4% formaldehyde, and permeabilized with 0.2% Triton X-100 (Junsei Chemical, 9002-93-1). After blocking with 1% BSA (Sigma-Aldrich, A5611), the cells were immunolabeled overnight at 4°C with primary antibodies: LC3B, MYC, TFEB, MTOR and LAMP2A (Abcam, ab18528). Lipid droplets were stained with BODIPY 493/503 (Thermo Fisher Scientific, D3922), and lysosomal activity was assessed by staining with LysoTracker Red (Thermo Fisher Scientific, L7528). Nuclei were stained with Hoechst (Thermo Fisher Scientific, 62249). Images were captured at 400x magnification using an LSM980 confocal microscope (Carl Zeiss AG, Oberkochen, Germany).

***Proximity ligation assay (PLA)***

Cells were fixed with 4% formaldehyde, permeabilized with 0.2% Triton X-100, and blocked with a blocking solution for 1 h. Primary antibodies against LAMTOR1 and MTOR were incubated overnight at 4°C. The PLA probe was added and incubated for 1 h at 37°C, followed by ligation for 30 min and amplification for 100 min at 37°C. Hoechst was used for nuclear staining, and red PLA puncta were visualized and quantified using an LSM980 confocal microscope (Carl Zeiss AG, Oberkochen, Germany).

***In silico docking study***

All molecular docking analyses were performed using Discovery Studio 2018 software (Accelrys Software Inc., San Diego, CA, USA). The 3D structure of LAMTOR1 (ID: Q9CQ22) was obtained from the AlphaFold protein structure database. For ligand docking, CDOCKER (a lattice-based molecular docking method using the CHARMM force field) was used and the binding energy (CDOCKER energy) was calculated.

***Animal experimental procedures***

The Institutional Animal Care and Use Committee of Yonsei University approved the study protocol (Permission number: IACUC-A-202110-1353-02). Mice were handled in accordance with the Institutional Animal Care and Use Committee and international guidelines for the ethical use of animals. Four-week-old male C57BL/6J mice (Raonbio Inc., Seoul, Republic of Korea) were purchased and acclimated for 2 weeks to a 12-h light/12-h dark cycle at 23±1°C and 40–60% humidity. Mice were fed either a normal chow diet (n = 4) or a choline-deficient, L-amino acid-defined, high-fat diet (CDAHFD) to induce MASH (n = 20) for 4 weeks. The CDAHFD-fed mice were further divided into two groups (n = 10 per group) that received vehicle or ACA (10 mg/kg), respectively, by intraperitoneal injection every two days for 4 weeks. Body weight of the mice was measured during the observation period. After the mice were sacrificed, serum and liver samples were collected, and tissue sections were prepared for histological analysis as described above.

***Measurement of serum AST and ALT levels***

GOT1/AST: glutamic-oxaloacetic transaminase 1 and GPT/ALT: glutamic--pyruvic transaminase levels were measured using a Cobas 8000 c502 analyzer (F. Hoffmann-La Roche AG, Basel, Switzerland) according to the recommended protocol of the colorimetric assay. Both GOT1/AST and GPT/ALT were measured according to IFCC standards (F. Hoffmann-La Roche AG, Basel, Switzerland).

***Histological analysis and immunohistochemistry***

Masson’s trichrome staining was performed (Obenbio, Inc., Seoul, Republic of Korea). Samples were analyzed using the CaseViewer software. For immunohistochemistry, tissues fixed in 10% formaldehyde (Sigma-Aldrich, 252549) were embedded in 15% sucrose (Ducksan Neolux Co., Ltd., 57-50-1) followed by 30% sucrose (Ducksan Neolux Co., Ltd., 57-50-1). The tissues were embedded in OCT (Sakura Finetek USA, Inc., 4583), sectioned, and mounted onto slides, frozen rapidly in liquid nitrogen, and blocked in 5% BSA and 0.3% Triton X-100 for 1 h. Slides were then incubated overnight at 4°C with 1% BSA and the following antibodies: ADGRE1/EMR1 (Bio-Rad Laboratories, MCA497), VMP1 (OriGene, TA351822), TFEB, p-PRKAA/AMPK, SQSTM1 (Becton, Dickinson and Company, BD610833), or LC3 (Medical & Biological Laboratories, PM036), The slides were then washed, incubated with secondary antibodies (Invitrogen, A11005, A11008) for 1 h at room temperature and mounted using mounting solution (Sigma-Aldrich, DUO82040) containing Hoechst. Images were captured at 400x magnification using an LSM980 confocal microscope (Carl Zeiss AG, Oberkochen, Germany).

***Statistical analysis***

All data are expressed as the mean ± standard error using GraphPad Prism (ver. 9.00 for Windows; GraphPad Software, Inc., San Diego, CA, USA). Data were obtained from at least three independent experiments. Statistical analyses were performed using unpaired, two-tailed Student’s t-test. P values less than 0.05 were considered statistically significant (* indicates p < 0.05, ** indicates p < 0.01, *** indicates p < 0.001, **** indicates p < 0.0001).

**Acknowledgements**

The authors thank Prof. Chang Hee Kang, Seoul National University, and Prof. Jaewhan Song, Yonsei University, for their constructive comments. This study was supported by the National Research Foundation of Korea and was funded by the Government of the Republic of Korea (MSIP; 2021R1A3B1077371), the Brain Korea 21 Plus Project of the Republic of Korea and ICONS (Institute of Convergence Science) and YFL (Yonsei Frontier Laboratory), supported by Yonsei University and was supported by a grant from the Korea Basic Science Institute (KBSI) (Research Grant No. A423200) and grant from the National Research Foundation of Korea (NRF) funded by the Ministry of Science and ICT of the Government of Korea (NRF2022M3H9A2096186 to J.Y. Kim)

**Disclosure statement**

No potential conflict of interest was reported by the author(s).

**Data availability statement**

The raw MS data files for total proteomics have been deposited to the repository MassIVE with identifier MSV000096568.

**References**

[1] Han SK, Baik SK, Kim MY. Non-alcoholic fatty liver disease: Definition and subtypes. Clin Mol Hepatol. 2023;29(Suppl):S5-S16. doi: 10.3350/cmh.2022.0424

[2] Perumpail BJ, Khan MA, Yoo ER, et al. Clinical epidemiology and disease burden of nonalcoholic fatty liver disease. World J Gastroenterol. 2017;23(47):8263-8276. doi: 10.3748/wjg.v23.i47.8263

[3] Riazi K, Raman M, Taylor L, et al. Dietary patterns and components in nonalcoholic fatty liver disease (NAFLD): what key messages can health care providers offer? Nutrients. 2019;11(12):2878. doi: 10.3390/nu11122878

[4] Enomoto H, Bando Y, Nakamura H, et al. Liver fibrosis markers of nonalcoholic steatohepatitis. World J Gastroenterol. 2015;21(24):7427-7435. doi: 10.3748/wjg.v21.i24.7427

[5] Hwang M-S, Baek J-H, Song J-K, et al. Tschimganidine reduces lipid accumulation through AMPK activation and alleviates high-fat diet-induced metabolic diseases. BMB Rep. 2023;56(4):246-251. doi: 10.5483/BMBRep.2022-0211

[6] Harvey BE. NASH: regulatory considerations for clinical drug development and US FDA approval. Acta Pharmacol Sin. 2022;43(5):1210-1214. doi: 10.1038/s41401-021-00832-z

[7] Chen C-L, Lin Y-C. Autophagy dysregulation in metabolic associated fatty liver disease: A new therapeutic target. Int J Mol Sci. 2022;23(17):10055. doi: 10.3390/ijms231710055

[8] Ding H, Ge G, Tseng Y, et al. Hepatic autophagy fluctuates during the development of non-alcoholic fatty liver disease. Ann Hepatol. 2020;19(5):516-522. doi: 10.1016/j.aohep.2020.06.001

[9] Byrnes K, Blessinger S, Bailey NT, et al. Therapeutic regulation of autophagy in hepatic metabolism. Acta Pharm Sin B. 2022;12(1):33-49. doi: 10.1016/j.apsb.2021.07.021

[10] Jang H-J, Lee YH, Dao T, et al. Thrap3 promotes nonalcoholic fatty liver disease by suppressing AMPK-mediated autophagy. Exp Mol Med. 2023;55(8):1720-1733. doi: 10.1038/s12276-023-01047-4

[11] Czaja MJ. Function of autophagy in nonalcoholic fatty liver disease. Dig Dis Sci. 2016;61:1304-1313. doi: 10.1007/s10620-015-4025-x

[12] Semwal RB, Semwal DK, Combrinck S, et al. Acacetin—A simple flavone exhibiting diverse pharmacological activities. Phytochem Lett. 2019;32(3):56-65. doi: 10.1016/j.phytol.2019.04.021

[13] Van De Wier B, Koek GH, Bast A, Haenen GR. The potential of flavonoids in the treatment of non-alcoholic fatty liver disease. Crit Rev Food Sci Nutr. 2017;57(4):834-855. doi: 10.1080/10408398.2014.952399

[14] Liou C-J, Wu S-J, Shen S-C, et al. Acacetin protects against non-alcoholic fatty liver disease by regulating lipid accumulation and inflammation in mice. Int J Mol Sci. 2022;23(9):4687. doi: 10.3390/ijms23094687

[15] Jiang Z, Sun H, Miao J, et al. The natural flavone acacetin protects against high-fat diet-induced lipid accumulation in the liver via the endoplasmic reticulum stress/ferroptosis pathway. Biochem Biophys Res Commun. 2023;640:183-191. doi: 10.1016/j.bbrc.2022.12.014

[16] Zhang H-W, Hu J-J, Fu R-Q, et al. Flavonoids inhibit cell proliferation and induce apoptosis and autophagy through downregulation of PI3Kγ mediated PI3K/AKT/mTOR/p70S6K/ULK signaling pathway in human breast cancer cells. Sci Rep. 2018;8(1):11255. doi: 10.1038/s41598-018-29308-7

[17] Liu J, Wang Y-G, Yu S-Y, et al. Protective effect of acacetin in human periodontal ligament cells via regulation of autophagy and inflammation. Pharmazie. 2020;75(9):436-439 doi: 10.1691/ph.2020.0435

[18] Hwang H-Y, Cho YS, Kim JY, et al. Autophagic inhibition via lysosomal integrity dysfunction leads to antitumor activity in glioma treatment. Cancers. 2020;12(3):543. doi: 10.3390/cancers12030543

[19] Kim TY, Ji ES, Lee JY, et al. DNA polymerase alpha subunit B is a binding protein for erlotinib resistance in non-small cell lung cancer. Cancers. 2020;12(9):2613. doi: 10.3390/cancers12092613

[20] Park SI, Cho SM, Atsumi S, et al. NDUFA12 As a functional target of the anticancer compound ertredin in human hepatoma cells as revealed by label-free chemical proteomics. J Proteomoe Res. 2023;23(1):130-141. doi: 10.1021/acs.jproteome.3c00471

[21] Tsujimoto K, Takamatsu H, Kumanogoh A. The Ragulator complex: delving its multifunctional impact on metabolism and beyond. Inflamm Regen. 2023;43(1):28. doi: 10.1186/s41232-023-00278-2

[22] Kimura T, Kumanogoh A, Okada M. Roles of Lamtor1 in Macrophages, CD4+ T-cells, and Regulatory T-cells. Crit Rev Immunol. 2018;38(5):403-414. doi: 10.1615/CritRevImmunol.2018028252

[23] Fang T, Wang H, Pan X, et al. Mouse models of nonalcoholic fatty liver disease (NAFLD): pathomechanisms and pharmacotherapies. Int J Biol Sci. 2022;18(15):5681-5697. doi: 10.7150/ijbs.65044

[24] Jiang X, Fulte S, Deng F, et al. Lack of VMP1 impairs hepatic lipoprotein secretion and promotes non-alcoholic steatohepatitis. J Hepatol. 2022;77(3):619-631. doi: 10.1016/j.jhep.2022.04.010

[25] Marwaha R, Sharma M. DQ-Red BSA trafficking assay in cultured cells to assess cargo delivery to lysosomes. Bio-Protocol. 2017;7(19):e2571-e2571. doi: 10.21769/BioProtoc.2571

[26] Kim D, Hwang H-Y, Kim JY, et al. FK506, an immunosuppressive drug, induces autophagy by binding to the V-ATPase catalytic subunit A in neuronal cells. J Proteomoe Res. 2017;16(1):55-64. doi: 10.1021/acs.jproteome.6b00638

[27] Nowosad A, Jeannot P, Callot C, et al. p27 controls Ragulator and mTOR activity in amino acid-deprived cells to regulate the autophagy–lysosomal pathway and coordinate cell cycle and cell growth. Nat Cell Biol. 2020;22(9):1076-1090. doi: 10.1038/s41556-020-0554-4

[28] Sanchez TW, Ronzetti MH, Owens AE, et al. Real-time cellular thermal shift assay to monitor target engagement. ACS Chem Biol. 2022;17(9):2471-2482. doi: 10.1021/acschembio.2c00334

[29] Kenny PW. Hydrogen-bond donors in drug design. J Med Chem. 2022;65(21):14261-14275. doi: 10.1021/acs.jmedchem.2c01147

[30] Zhang C-S, Jiang B, Li M, et al. The lysosomal v-ATPase-Ragulator complex is a common activator for AMPK and mTORC1, acting as a switch between catabolism and anabolism. Cell Metab. 2014;20(3):526-540. doi: 10.1016/j.cmet.2014.06.014

[31] Zoncu R, Bar-Peled L, Efeyan A, et al. mTORC1 senses lysosomal amino acids through an inside-out mechanism that requires the vacuolar H+-ATPase. Science. 2011;334(6056):678-683. doi: 10.1126/science.1207056

[32] Narita M, Inoki K. Rags connect mTOR and autophagy. Small GTPases. 2012;3(2):111-114. doi: 10.4161/sgtp.19422

[33] Chao X, Wang S, Zhao K, et al. Impaired TFEB-mediated lysosome biogenesis and autophagy promote chronic ethanol-induced liver injury and steatosis in mice. Gastroenterology. 2018;155(3):865-879. e12. doi: 10.1053/j.gastro.2018.05.027

[34] Paquette M, El-Houjeiri L, C. Zirden L, et al. AMPK-dependent phosphorylation is required for transcriptional activation of TFEB and TFE3. Autophagy. 2021;17(12):3957-3975. doi: 10.1080/15548627.2021.1898748

[35] Wang S, Ni H-M, Chao X, et al. Impaired TFEB-mediated lysosomal biogenesis promotes the development of pancreatitis in mice and is associated with human pancreatitis. Autophagy. 2019;15(11):1954-1969. doi: 10.1080/15548627.2019.1596486

[36] Martina JA, Chen Y, Gucek M, Puertollano R. MTORC1 functions as a transcriptional regulator of autophagy by preventing nuclear transport of TFEB. Autophagy. 2012;8(6):903-914. doi: 10.4161/auto.19653

[37] Müller FA, Sturla SJ. Human in vitro models of nonalcoholic fatty liver disease. Curr Opin Toxicol. 2019;16:9-16. doi: 10.1016/j.cotox.2019.03.001

[38] Suppli MP, Rigbolt KT, Veidal SS, et al. Hepatic transcriptome signatures in patients with varying degrees of nonalcoholic fatty liver disease compared with healthy normal-weight individuals. Am J Physiol Gastrointest Liver Physiol. 2019;316(4):G462-G472. doi: 10.1152/ajpgi.00358.2018

[39] Lu Y-C, Chang C-C, Wang C-P, et al. Circulating fatty acid-binding protein 1 (FABP1) and nonalcoholic fatty liver disease in patients with type 2 diabetes mellitus. Int J Med Sci. 2020;17(2):182-190. doi: 10.7150/ijms.40417

[40] Caballero F, Fernández A, De Lacy AM, et al. Enhanced free cholesterol, SREBP-2 and StAR expression in human NASH. J Hepatol. 2009;50(4):789-796. doi: 10.1016/j.jhep.2008.12.016

[41] Min H-K, Kapoor A, Fuchs M, et al. Increased hepatic synthesis and dysregulation of cholesterol metabolism is associated with the severity of nonalcoholic fatty liver disease. Cell Metab. 2012;15(5):665-674. doi: 10.1016/j.cmet.2012.04.004

[42] Mukai T, Egawa M, Takeuchi T, et al. Silencing of FABP 1 ameliorates hepatic steatosis, inflammation, and oxidative stress in mice with nonalcoholic fatty liver disease. FEBS Open Bio. 2017;7(7):1009-1016. doi: 10.1002/2211-5463.12240

[43] Niture S, Lin M, Rios-Colon L, et al. Emerging roles of impaired autophagy in fatty liver disease and hepatocellular carcinoma. Int J Hepatol. 2021:6675762. doi: 10.1155/2021/6675762

[44] Ren Y-S, Li H-L, Piao X-H, et al. Drug affinity responsive target stability (DARTS) accelerated small molecules target discovery: Principles and application. Biochem Pharmacol. 2021;194:114798. doi: 10.1016/j.bcp.2021.114798

[45] Mu Z, Wang L, Deng W, et al. Structural insight into the Ragulator complex which anchors mTORC1 to the lysosomal membrane. Cell Discov. 2017;3:17049. doi: 10.1038/celldisc.2017.49

[46] Yonehara R, Nada S, Nakai T, et al. Structural basis for the assembly of the Ragulator-Rag GTPase complex. Nat Commun. 2017;8(1):1625. doi: 10.1038/s41467-017-01762-3

[47] Panwar V, Singh A, Bhatt M, et al. Multifaceted role of mTOR (mammalian target of rapamycin) signaling pathway in human health and disease. Signal Transduct Target Ther. 2023;8(1):375. doi: 10.1038/s41392-023-01608-z

[48] Caccamo A, Magrì A, Medina DX, et al. mTOR regulates tau phosphorylation and degradation: implications for Alzheimer's disease and other tauopathies. Aging cell. 2013;12(3):370-380. doi: 10.1111/acel.12057

[49] Zou Z, Tao T, Li H, Zhu X. mTOR signaling pathway and mTOR inhibitors in cancer: progress and challenges. Cell Biosci. 2020;10(1):31. doi: 10.1186/s13578-020-00396-1

[50] Sarbassov DD, Ali SM, Sengupta S, et al. Prolonged rapamycin treatment inhibits mTORC2 assembly and Akt/PKB. Mol Cell. 2006;22(2):159-168. doi: 10.1016/j.molcel.2006.03.029

[51] Xie J, Herbert TP. The role of mammalian target of rapamycin (mTOR) in the regulation of pancreatic β-cell mass: implications in the development of type-2 diabetes. Cell Mol Life Sci. 2012;69(8):1289-1304. doi: 10.1007/s00018-011-0874-4

[52] Carracedo A, Ma L, Teruya-Feldstein J, et al. Inhibition of mTORC1 leads to MAPK pathway activation through a PI3K-dependent feedback loop in human cancer. J Clin Invest. 2008;118(9):3065-3074. doi: 10.1172/JCI34739

[53] Rozengurt E, Soares HP, Sinnet-Smith J. Suppression of feedback loops mediated by PI3K/mTOR induces multiple overactivation of compensatory pathways: an unintended consequence leading to drug resistance. Mol Cnacer Ther. 2014;13(11):2477-2488. doi: 10.1158/1535-7163.MCT-14-0330

[54] Carvalho PC, Xu T, Han X, et al. YADA: a tool for taking the most out of high-resolution spectra. Bioinformatics. 2009;25(20):2734-2736. doi: 10.1093/bioinformatics/btp489

[55] Tabb DL, McDonald WH, Yates JR. DTASelect and Contrast: tools for assembling and comparing protein identifications from shotgun proteomics. J Proteomoe Res. 2002;1(1):21-26. doi: 10.1021/pr015504q

[56] Raso C, Cosentino C, Gaspari M, et al. Characterization of breast cancer interstitial fluids by TmT labeling, LTQ-Orbitrap Velos mass spectrometry, and pathway analysis. J Proteomoe Res. 2012;11(6):3199-3210. doi: 10.1021/pr2012347

**Figure Legends**

**Figure 1.** Acacetin ameliorates pathological liver damage in the CDAHFD-induced MASH mouse model. (**A**) Overview of the CDAHFD-induced mouse model experiment. (**B**) Overall scheme of the experiment. (**C**) External photographs of representative livers from chow-fed mice (n = 4), CDAHFD-fed mice (n = 10), and CDAHFD-fed mice treated with 10 mg/kg ACA (n = 10). Mice were injected intraperitoneally with ACA for 4 weeks. Scale bar: 10 mm. (**D**) Body weight of the mice. (**E**) Liver weight of the mice. ns: not significant (**F and G**) Serum levels of GOT1/AST and GPT/ALT in each group of mice. Values are means ± SEM; n=10, *p < 0.05, ****p < 0.0001. (**H**) Representative images of neutral triglycerides and lipids in mouse liver tissues examined by light microscopy after Oil Red O staining. Scale bar: 10 µm. (**I**) Representative images of Masson’s trichrome staining in liver sections from each group. Scale bar: 100 µm. (**J**) Quantitative data of Masson’s trichrome staining of relative fibrotic area (red allow: fibrosis, black arrow: vacuolation). Values are means ± SEM; ****p < 0.0001. (**K**) Representative images of immunostaining for ADGRE1/EMR1 macrophage antigens (red) in liver. Scale bar: 20 µm. (**L**) Representative images of immunostaining for VMP1 expression (yellow) in mouse liver tissues. Scale bar: 20 µm. (**M**) Quantitative data of ADGRE1/EMR1 expression. Values are means ± SEM; **p < 0.01, ***p < 0.001. (**N**) Quantitative data of VMP1 expression. Values are means ± SEM; **p < 0.01.

**Figure 2.** Acacetin inhibits lipid accumulation in an autophagy-dependent manner. (**A**) Effect of ACA on cell proliferation using MTT assay in 3T3-L1 cells. (**B**) Effect of ACA on cell cytotoxicity using trypan blue assay in 3T3-L1 cells. Values are means ± SEM; n > 30 cells. *p < 0.05, ns: not significant. (**C**) Effect of ACA on cell proliferation using MTT assay in HepG2 cells. (**D**) Effect of ACA on cell cytotoxicity using trypan blue assay in HepG2 cells. Values are means ± SEM; n > 30 cells. *p < 0.05, **p < 0.01, ***p < 0.001, ns: not significant. (**E**) Representative image of Oil Red O staining. 3T3-L1 cells were differentiated for 8 days and treated with ACA (50 μM) every 2 days from day 4 to day 8. Cells were stained with Oil Red O and examined microscopically. Scale bar: 50 µm. (**F**) Oil Red O dye was extracted, and the extracted dye solution was measured for absorbance at 490 nm. Values are means ± SEM; ****p < 0.0001. (**G**) 3T3-L1 cells were incubated in DMEM containing DQ-BSA (10 μg/mL) for 2 h. The cells were then washed with PBS, treated with Rapa (10 μM), bafilomycin A_1_ (10 nM) or ACA (50 μM) for 6 h, and fixed, and the fluorescence of DQ-BSA (red) was imaged using a confocal microscope. Scale bar: 20 µm. (**H**) Quantitative data of lysosomal puncta of DQ-BSA. Values are means ± SEM; ****p < 0.0001. (**I**) Representative images of autophagic flux evaluation using mRFP-GFP-MAP1LC3B/LC3 in the presence of each compound. HepG2 cells were treated with Rapa (10 μM), bafilomycin A_1_ (10 nM) or ACA (50 μM) for 24 h. Scale bar: 10 µm. (**J**) Quantitative data of mRFP-GFP-MAP1LC3B/LC3. Values are means ± SEM; n > 10 cells, *p < 0.05, ****p < 0.0001. (**K**) Measurement of time-dependent expression of autophagy markers (LC3 and SQSTM1) using western blot. The differentiated 3T3-L1 cells were treated with Rapa (10 μM) or ACA (30 μM or 50 μM) for 24 h, 48 h, or 72 h. (**L**) The differentiated 3T3-L1 cells were co-treated with ACA (50 μM) for 24 h in the absence or presence of the autophagy inhibitor CQ (10 μM). (**M**) The differentiated 3T3-L1 cells were treated with ACA (50 μM) in the absence or presence of the autophagy inhibitor CQ (10 μM). Cells were subjected to immunostaining with an anti-LC3 antibody (red) and stained with BODIPY 493/503 (green) to visualize lipid droplets. Confocal microscopy was used to examine the colocalization of LC3 and BODIPY 493/503 (yellow). Scale bar: 20 µm. (**N**) Quantitative data of lipid droplet intensity. Values are means ± SEM; **p < 0.01. (**O**) HeLa WT cells were treated with ACA (50 μM) for 24 h, followed by staining with BODIPY 493/503 (green) to visualize lipid droplets. Lipid droplets were analyzed using confocal microscopy. Scale bar: 20 µm. (**P**) Quantitative data of lipid droplet intensity. Values are means ± SEM; ****p < 0.0001. (**Q**) HeLa *ATG3* KO cells were treated with ACA (50 μM) for 24 h, followed by staining with BODIPY 493/503 (green) to visualize lipid droplets. Lipid droplets were analyzed using confocal microscopy. Scale bar: 20 µm. (**R**) Quantitative data of lipid droplet intensity. Values are means ± SEM; ns: not significant.

**Figure 3.** Identification of the pharmacological target of acacetin by DARTS LC-MS/MS analysis. (**A**) Flowchart of DARTS LC-MS/MS to identify the protein target of ACA. First, pronase digestion was performed and DARTS analysis was used to identify the target proteins of ACA in the cell lysate proteome pool. Second, DARTS analysis was used to stabilize target proteins bound to ACA by structural conformational change, and proteins that became resistant to pronase degradation were analyzed and identified by LC-MS/MS. Finally, LAMTOR1 was selected from the 18 protein candidates with pronase resistance, an increase in binding stability of more than 15%, and reasonable sequence coverage. (**B**) Functional enrichment heatmap analysis of the 18 candidate proteins. (**C**) STRING analysis of protein candidates selected by DARTS LC-MS/MS analysis, which is a label-free method. (**D**) Pronase-dependent DARTS analysis was performed for target validation. 3T3-L1 cell lysate was treated with vehicle control or ACA (50 μM). Compound binding was measured for 3 h at 4℃, followed by treatment with 1 μg/mL or 2.5 μg/mL for 10 min. (**E**) Quantitative data of the pronase-dependent DARTS analysis. (**F**) Dose-dependent DARTS analysis was conducted for target validation. 3T3-L1 cell lysate was treated with vehicle control or ACA at concentrations ranging from 10 μM to 200 μM. Binding of the compound was performed at 4℃ for 3 h, followed by pronase treatment with 1 μg/mL for 10 min. (**G**) Quantitative data of the dose-dependent DARTS analysis.

**Figure 4.** Analysis of the binding site of acacetin to LAMTOR1 by *in silico* docking***.*** (**A**) *In silico* docking analysis of ACA directly interacting with LAMTOR1. (**B**) Two-dimensional diagram of the binding interaction and predicted amino acid binding sites of LAMTOR1 with ACA. (**C**) HEK293 cells were transfected with WT MYC-LAMTOR1, MYC-LAMTOR1^L22A^, MYC-LAMTOR1^D24G^, or MYC-LAMTOR1^D49A^ vectors for 48 h. Cell lysate was treated with vehicle control or ACA (50 μM), and compound binding was assessed at 4℃ for 3 h, followed by pronase treatment with 1 μg/mL for 10 min. (**D**) Quantitative data of the point mutation DARTS analysis. Values are means ± SEM; *p < 0.05, **p < 0.01, ns: not significant. (**E**) HEK293 cells were transfected with WT MYC-LAMTOR1, MYC-LAMTOR1^L22A^, MYC-LAMTOR1^D24G^, or MYC-LAMTOR1^D49A^ vectors for 48 h, treated with vehicle control or ACA (50 μM), fixed, and immunostained with MYC (red) and TFEB (green). Images were acquired by confocal microscopy. Scale bar: 20 µm. (**F**) HEK293 cells were transfected with WT MYC-LAMTOR1, MYC-LAMTOR1^L22A^, MYC-LAMTOR1^D24G^, or MYC-LAMTOR1^D49A^ vectors for 48 h, treated with vehicle control or ACA (50 μM), fixed, and immunostained with MYC (red) and LC3 (green); Images were acquired by confocal microscopy. Scale bar: 20 µm. (**G**) Quantification of relative nuclear TFEB intensity per cell. Values are means ± SEM; **p < 0.01, ****p < 0.0001, ns: not significant. (**H**) Quantification of the number of LC3 puncta per cell. Values are means ± SEM; *p < 0.05, **p < 0.01, ***p < 0.001.

**Figure 5.** Acacetin induces TFEB nuclear translocation by modulating the MTORC1/AMPK axis. (**A**) Schematic illustration of the regulation of MTORC1 by the LAMTOR complex. (**B**) 3T3-L1 cells were treated with vehicle control or ACA (50 μM) for 4 h. PLA analysis was performed to assess the interaction between MTOR and LAMTOR1. Scale bar: 20 µm. (**C**) Quantitative data of the PLA analysis representing the interaction between MTOR and LAMTOR1. Values are means ± SEM; n > 30 cells. ****p < 0.0001. (**D**) The differentiated 3T3-L1 cells were treated with vehicle control or ACA (50 μM) for 4 h. Immunostaining was performed for MTOR (red) and LAMP2 (green), followed by confocal microscopy. Scale bar: 10 µm. (**E**) Manders' coefficient values for the colocalization of MTOR (red) and LAMP2 (green) in 3T3-L1 cells [M1: LAMP2 (green) overlapping with MTOR (red), M2: MTOR (red) overlapping with LAMP2 (green)]. Values are means ± SEM; **p < 0.01. (**F**) The differentiated 3T3-L1 cells were treated with vehicle control or ACA (50 μM) for 24 h. The protein expression levels of p-MTOR and MTOR were analyzed by western blot analysis. (**G**) The differentiated 3T3-L1 cells were treated with vehicle control or ACA (30 μM, 50 μM) in the absence or presence of the AMPK inhibitor compound C (20 μM) for 24 h. The protein expression levels of p-PRKAA/AMPK and AMPK were analyzed by western blot analysis. (**H**) 3T3-L1 cells were treated with vehicle control or ACA (50 μM) for 3 h, and nuclear fractionation was followed by western blot analysis.

**Figure 6.** Acacetin restores autophagy in the MASH mouse model. (**A**) PLA analysis of MTOR and LAMTOR1 in liver tissues of mice treated with vehicle or ACA (10 mg/kg). Scale bar: 20 µm. (**B**) Representative images of immunostaining for p-PRKAA/AMPK expression (green) in mouse liver tissues. Scale bar: 50 µm. (**C**) Quantitative data of p-PRKAA/AMPK expression. Values are means ± SEM; ***p < 0.001. (**D**) Representative images of TFEB (green) immunostaining from the cytosol to the nucleus in liver tissues of mice treated with vehicle or ACA (10 mg/kg). Arrows indicate the translocation of TFEB from the cytosol to the nucleus. Scale bar: 20 µm. (**E**) Representative images of immunostaining for SQSTM1 expression (red) in mouse liver tissues. Scale bar: 20 µm. (**F**) Quantitative data of SQSTM1 expression. Values are means ± SEM; ****p < 0.0001. (**G**) Representative image of immunostaining for LC3 (red) and BODIPY 493/503 (green). Confocal images were examined for colocalization of LC3 and BODIPY 493/503 (yellow). Scale bar: 20 µm.

**Figure 7.** LAMTOR1 inhibition contributes to lipid reduction via autophagy and is as an important factor in MAFLD and MASH patients. (**A**) 3T3-L1 cells were either untreated or transfected with *Lamtor1*-targeting shRNA for 48 h. Protein levels of p-MTOR, MTOR, p-RPS6KB, and RPS6KB were analyzed by western blot analysis. (**B**) 3T3-L1 cells were either untreated or transfected with *Lamtor1*-targeting shRNA for 48 h, followed by nuclear fractionation and western blot analysis. (**C**) 3T3-L1 cells were either untreated or transfected with *Lamtor1*-targeting shRNA for 48 h. LysoTracker staining (red) was performed and confirmed by confocal microscopy. Scale bar: 20 µm. (**D**) Quantitative data of lysosomal fluorescence intensity from LysoTracker per cell. Values are means ± SEM; n > 30 cells. ****p < 0.0001. (**E**) Representative images of autophagic flux evaluation using mRFP-GFP-MAP1LC3B/LC3 in the presence or absence of *LAMTOR1*-targeting shRNA transfection for 48 h in HepG2 cells. Scale bar: 10 µm. (**F**) Quantitative data of mRFP-GFP-MAP1LC3B/LC3. Values are means ± SEM; n > 10 cells, **p < 0.01, ****p < 0.0001. (**G**) Representative image of Oil Red O staining. 3T3-L1 cells were either untreated or transfected with LAMTOR1-targeting shRNA for 48 h. and then differentiated for 8 days. Scale bar: 2 µm. (**H**) RNA sequencing analysis of LAMTOR1 expression in MAFLD and MASH patient cohorts from the Gene Expression Omnibus (GEO: GSE126848). Values are means ± SEM; *p < 0.05, ***p < 0.001. (**I** and **J**) Significant positive correlations between *LAMTOR1* and MAFLD-related factors (*SREBF2*, *FABP1*) in the GEO dataset (GSE126848). (**K**) Summary of the study on LAMTOR1-mediated autophagy regulation by ACA treatment: In metabolic dysfunction associated fatty liver disease, abnormal autophagy operation causes autophagy dysfunction. ACA binds to the target protein LAMTOR1 and activates autophagy through the inhibition of LAMTOR1. ACA induces the translocation of TFEB to the nucleus by inhibiting MTORC1, that ameliorates autophagy dysfunction in metabolic disease.
