# Supplementary material for: Inhibition of lysosomal LAMTOR1 increases autophagy by suppressing the MTORC1 pathway to ameliorate lipid accumulations in MAFLD
Source: Autophagy. 2025 Jul 6;21(12):2633–49. doi: 10.1080/15548627.2025.2519054 (PMC12758200; doi:10.1080/15548627.2025.2519054)
Supplement: Supplementary Material_not for review_R3_250528.docx [file KAUP_A_2519054_SM3936.docx]

**Supplemental material**

**Inhibition of Lysosomal LAMTOR1 Increases Autophagy by Suppressing the MTORC1 Pathway to Ameliorate Lipid Accumulations in MAFLD**


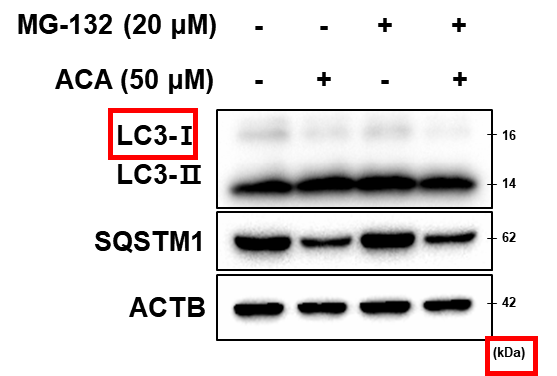


**Figure S1.** Acacetin acts independently of the ubiquitin-proteome pathway. The differentiated 3T3-L1 cells were co-treated with ACA (50 μM) for 24 h in the absence or presence of the proteasome inhibitor MG-132 (20 μM).

**
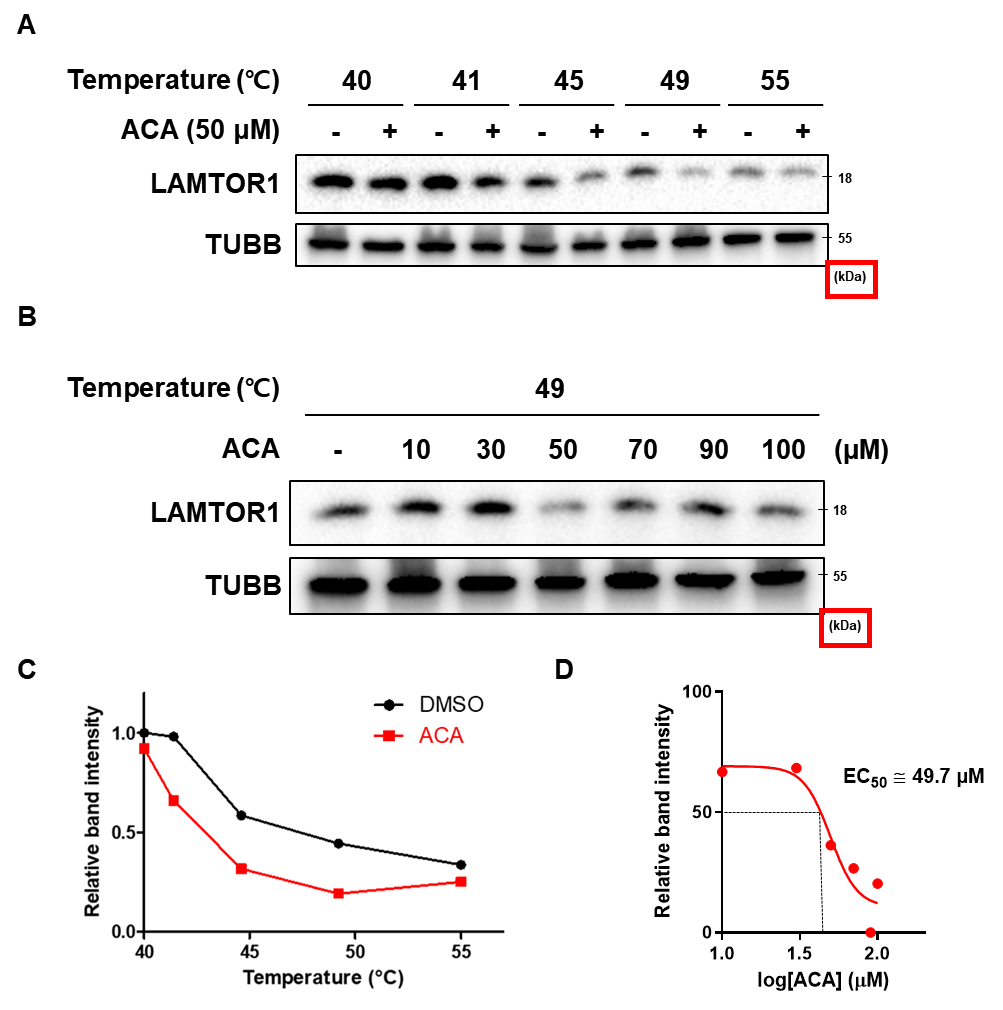
**

**Figure S2.** The CETSA for target protein identification and validation of acacetin in living cells. (**A**) CETSA-based identification of binding between ACA (50 μM) and LAMTOR1. Live cells were treated with vehicle control or ACA (50 μM) for 1 h, followed by a temperature shift. (**B**) The iso-thermal shift assay at 49℃ in living cells. Living cells were treated for 1 h with vehicle or ACA at concentrations ranging from 10 μM to 100 μM. (**C**) Quantitative data of the relative intensity of LAMTOR1 protein versus increased temperature. (**D**) IsoCETSA melting curve of ACA.

**
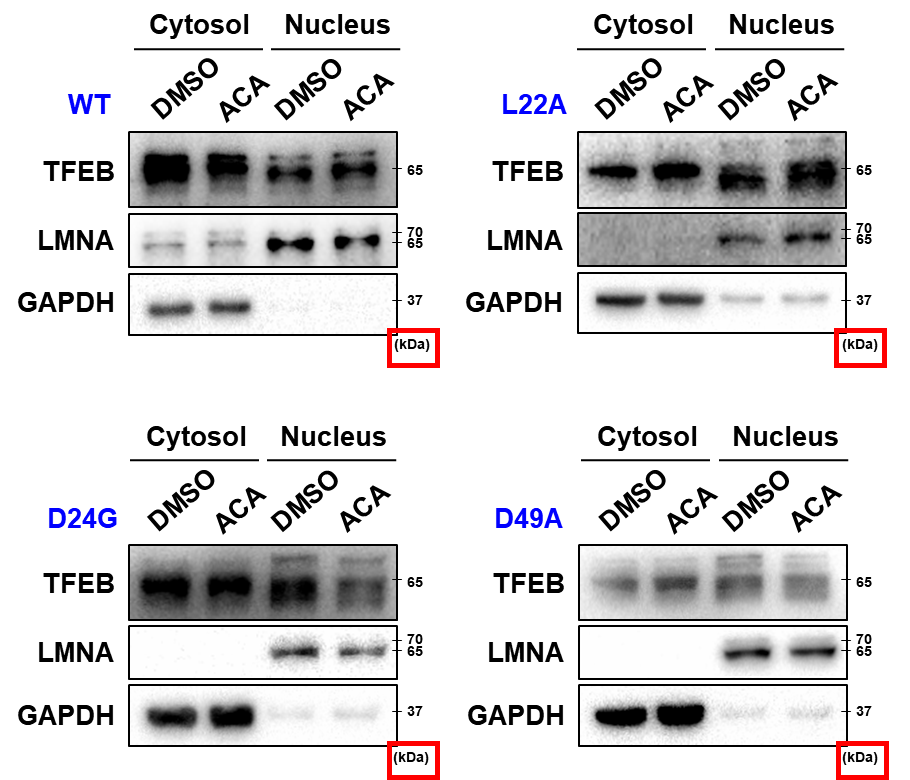
**

**Figure S3.** HEK293 cells were transfected with WT MYC-LAMTOR1, MYC-LAMTOR1^L22A^, MYC-LAMTOR1^D24G^, or MYC-LAMTOR1^D49A^ vectors for 48 h, followed by treatment with vehicle control or ACA (50 μM). Nuclear fractionation was performed, followed by western blot analysis.

**
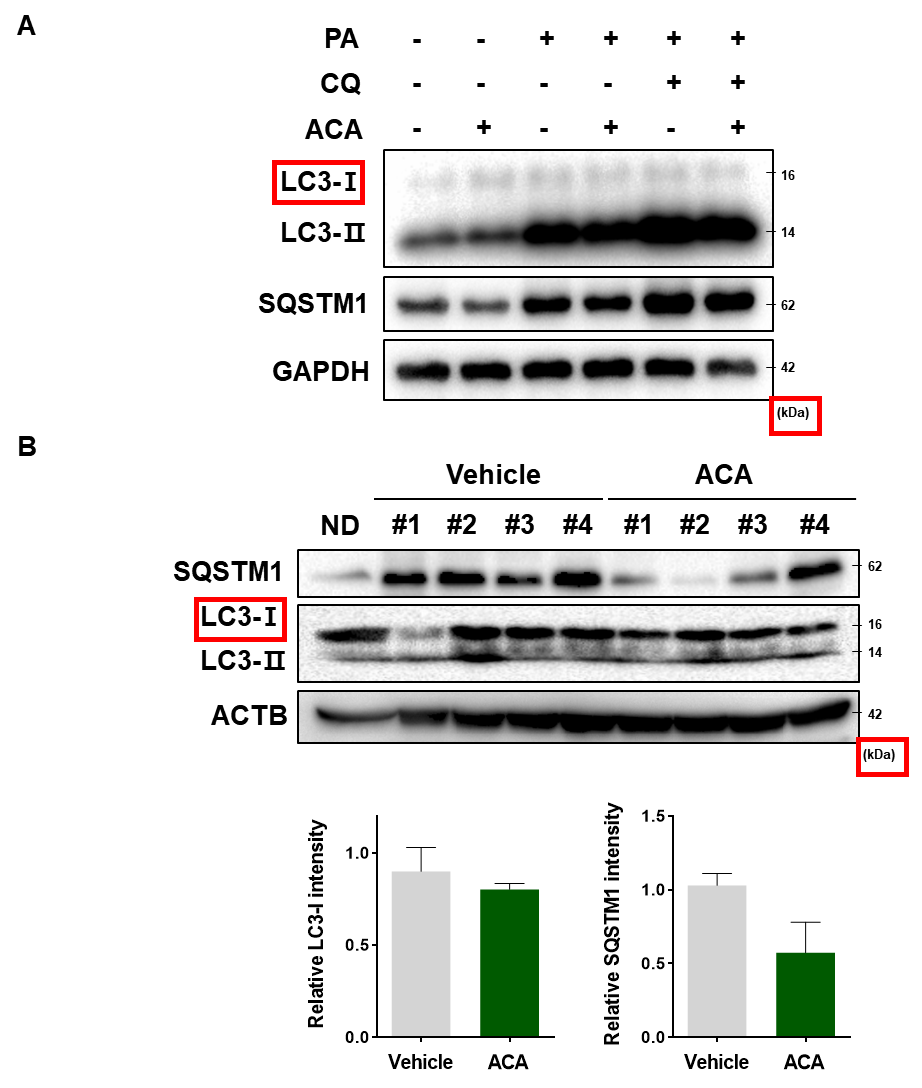
**

**Figure S4.** Expression of autophagy markers in *in vitro* and *in vivo* model of metabolic dysfunction-associated fatty liver disease. (**A**) HepG2 cells were treated with ACA (50 μM) alone or pretreated with PA (500 μM) for 24 h, followed by an additional 24 h treatment with ACA (50 μM). To assess autophagic flux, a group of PA-treated cells was further treated with CQ (10 μM) and ACA (50 μM) simultaneously for 24 h. The expression of autophagy markers was confirmed by western blot. (**B**) Liver tissues from mice treated with vehicle or ACA (10 mg/kg) were analyzed by western blot for autophagy markers SQSTM1 and LC3.


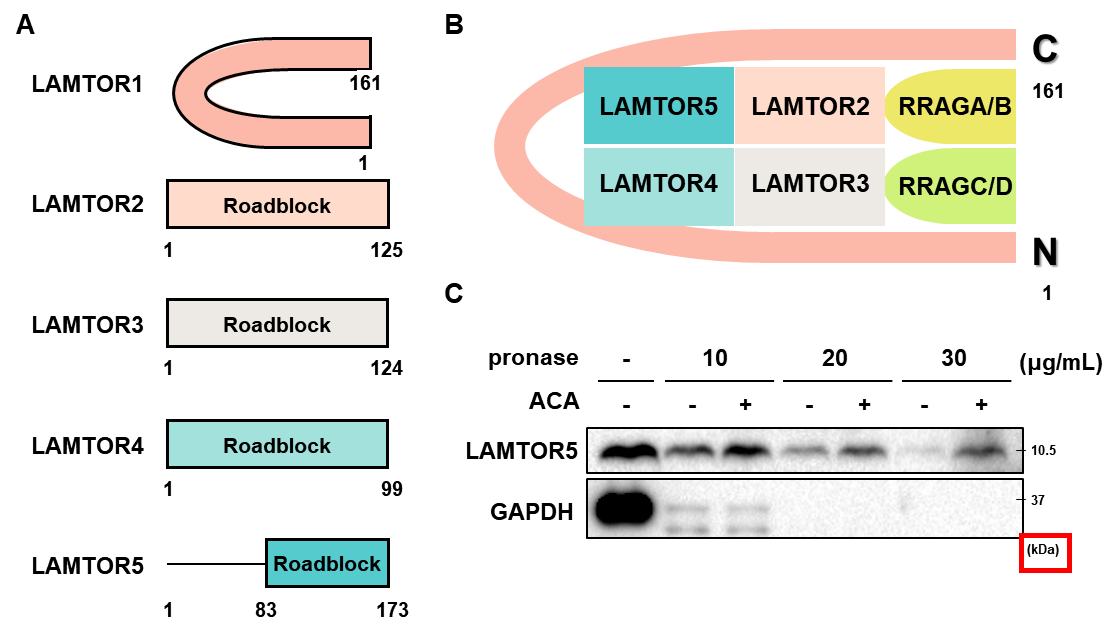


**Figure S5**. Structural analysis of the Ragulator complex and validation study of target protein candidates. (**A**) Domain arrangement of the Ragulator (LAMTOR1 to LAMTOR5) complex. (**B**) Schematic diagram of the 2D structure of the Ragulator-RRAG complex. (**C**) Pronase-dependent DARTS analysis was performed to validate target protein candidates. 3T3-L1 cell lysate was treated with control or ACA (50 μM), and compound binding was measured for 3 h at 4℃, followed by pronase treatment with 10 μg/mL to 30 μg/mL for 10 min.
