# Supplementary material for: Inhibition of lysosomal LAMTOR1 increases autophagy by suppressing the MTORC1 pathway to ameliorate lipid accumulations in MAFLD
Source: Autophagy. 2025 Jul 6;21(12):2633–49. doi: 10.1080/15548627.2025.2519054 (PMC12758200; doi:10.1080/15548627.2025.2519054)
Supplement: Figure abstract_250528.pdf [file KAUP_A_2519054_SM3935.pdf]

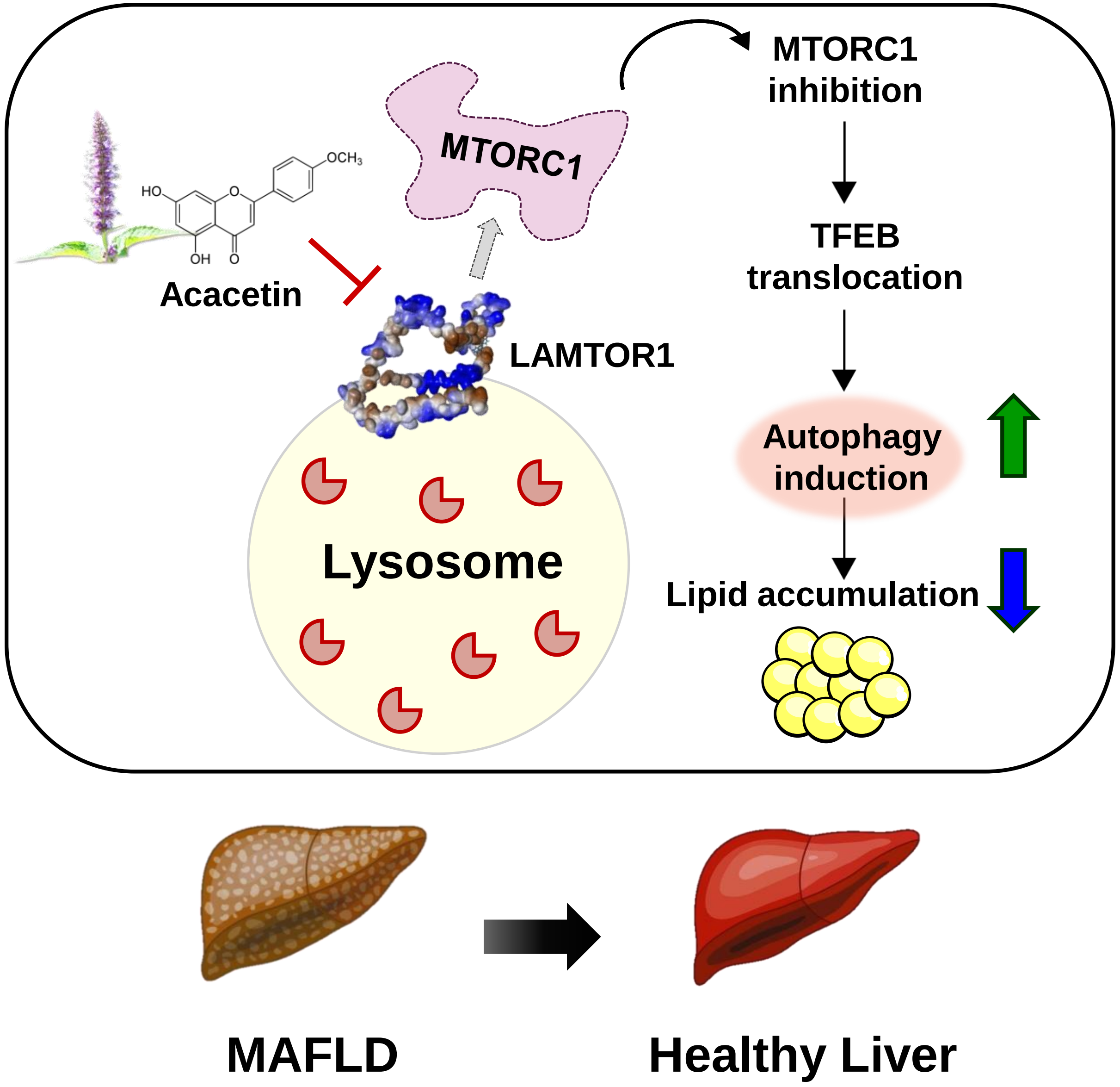

Lysosomal LAMTOR1 Responsible for Amelioration of Lipid Accumulation in MAFLD by Acacetin
